# Serological response following COVID-19 vaccines in patients living with HIV: a dose–response meta-analysis

**DOI:** 10.1038/s41598-023-37051-x

**Published:** 2023-06-19

**Authors:** Qian Zhou, Furong Zeng, Yu Meng, Yihuang Liu, Hong Liu, Guangtong Deng

**Affiliations:** 1 Department of Dermatology, Hunan Engineering Research Center of Skin Health and Disease, Hunan Key Laboratory of Skin Cancer and Psoriasis, Xiangya Hospital, Central South University, Changsha, 410008 Hunan China; 2 National Clinical Research Center for Geriatric Disorders, Xiangya Hospital, Central South University, Changsha, 410008 Hunan China; 3 Department of Oncology, Xiangya Hospital, Central South University, Changsha, 410008 Hunan China

**Keywords:** HIV infections, Viral infection, Vaccines

## Abstract

To quantify the pooled rate and risk ratio of seroconversion following the uncomplete, complete, or booster dose of COVID-19 vaccines in patients living with HIV. PubMed, Embase and Cochrane library were searched for eligible studies to perform a systematic review and meta-analysis based on PRIMSA guidelines. The pooled rate and risk ratio of seroconversion were assessed using the Freeman-Tukey double arcsine method and Mantel–Haenszel approach, respectively. Random-effects model was preferentially used as the primary approach to pool results across studies. A total of 50 studies involving 7160 patients living with HIV were analyzed. We demonstrated that only 75.0% (56.4% to 89.9%) patients living with HIV achieved a seroconversion after uncomplete vaccination, which improved to 89.3% (84.2% to 93.5%) after complete vaccination, and 98.4% (94.8% to 100%) after booster vaccination. The seroconversion rates were significantly lower compared to controls at all the stages, while the risk ratios for uncomplete, complete, and booster vaccination were 0.87 (0.77 to 0.99), 0.95 (0.92 to 0.98), and 0.97 (0.94 to 0.99), respectively. We concluded that vaccine doses were associated with consistently improved rates and risk ratios of seroconversion in patients living with HIV, highlighting the significance of booster vaccination for patients living with HIV.

## Introduction

Patients living with HIV are at high risk for severe coronavirus disease 2019 (COVID-19), with higher rates of hospitalization and mortality due to immunosuppression, other comorbidities, or social determinants of health^1–4^. COVID-19 vaccines have been found to be the main measure of reducing the severity and mortality of COVID-19 patients in clinical trials and real-world populations^5–8^. Therefore, patients living with HIV were an early priority group for vaccine eligibility. However, the fact that patients living with HIV have a reduced serological response to multiple vaccines, such as hepatitis B and seasonal influenza vaccines, compared to HIV-negative individuals has raised concerns about the efficacy of COVID-19 vaccination for patients living with HIV^9,10^.

A growing number of studies have reported serological responses between HIV-infected and non-HIV-infected patients. However, the conclusions were not consistent. For example, Madhi et al. showed that patients living with HIV achieved similar immunogenicity compared to healthy controls after uncomplete, and complete COVID-19 vaccine^11^. Bergman et al. demonstrated that SARS-CoV-2-naive patients living with HIV had attenuated humoral immune responses to COVID-19 vaccine compared with HIV-negative vaccine counterparts^12^. A meta-analysis has been performed to compare the serological response between HIV-infected and non-HIV-infected patients after a second dose of COVID-19 vaccine, but the analyses on uncompleted and booster doses of COVID-19 vaccine, as well as the exact seroconversion rate in patients living with HIV, were not further evaluated^13^. To a large extent, there is a sparsity of evidence on the serological response following COVID-19 vaccines in patients living with HIV.

Therefore, it is necessary to perform a meta-analysis of available evidence to quantify the pooled rate and risk ratio of seroconversion following the uncomplete, complete, or booster dose of COVID-19 vaccines in patients living with HIV.

## Methods

### Search strategy

This systematic review and meta-analysis was based on the Preferred Reporting Items for Systematic Reviews and Meta-analyses (PRIMSA) guidelines^14^ and was registered on PROSPERO with the registration number CRD42022359603. PubMed, Embase and Cochrane Library databases were searched from inception to 13th, September 2022 using the following terms: “COVID-19” OR “SARS-Cov-2” AND “vaccines” AND “HIV”. The full details of search strategies were provided in Supplementary Table S1.

### Inclusion and exclusion criteria

Study selection was conducted in three steps: removing the initial de-duplication, screening titles and abstracts, and reviewing the full text for eligible articles. Two researchers (Q.Z and F.Z.) independently evaluated eligibility, and discrepancies were solved by a third investigator (G.D.). Studies were included for analysis if they are cohort studies or randomized controlled trials that reported the seroconversion rate following the uncomplete, complete, or booster dose of COVID-19 vaccines in patients living with HIV; or provided risk ratios (RRs) for seroconversion and antibody titers following the uncomplete, complete, or booster dose of COVID-19 vaccines between HIV-infected and non-HIV-infected patients. Cohort studies were defined as those that sampled participants based on exposure, followed-up participants over time, and ascertained the outcomes^15^. The definition of seroconversion differed across studies, and Supplementary Table S2 provides the corresponding definition for each study. Uncomplete vaccination was defined as one dose of an mRNA vaccine (BNT162b2 or mRNA-1273), inactivated vaccine (BBIBP-CorV, Corona Vac, or Sinopharm), adenovirus vaccine (ChA-dOx1 nCoV-19), or recombinant protein vaccine (NVX-CoV2373). Complete vaccination was defined as two doses of an mRNA vaccine (BNT162b2 or mRNA-1273), inactivated vaccine (BBIBP-CorV, Corona Vac, or Sinopharm), or adenovirus vaccine (ChA-dOx1 nCoV-19), recombinant protein vaccine (NVX-CoV2373), or a single dose of adenovirus vaccine (Ad.26.COV2.S). Booster vaccination was defined as an additional shot after complete vaccination scheme. Studies on non-comparative cohorts with less than 10 participants were excluded. Case reports, case series, and studies with data inaccessible from the corresponding author were excluded. For multiple articles that reported identical outcomes from the same cohort, we selected those with the largest and most up-to-date studies.

### Data abstraction and quality assessment

Two investigators (Q.Z and F.Z) independently extracted data based on a predetermined proforma in Microsoft Excel. The following information was collected, including first author, publication year, country, study type, data source, patient number, control number, age, sex, vaccine type, vaccine dose, antiretroviral therapy, COVID-19 history, duration of follow-up, immunoassay, threshold for positive response, antibody titers, and adjustment parameters. We assessed risk of bias using two domain-based tools, including the Risk of Bias in Nonrandomized Studies of Interventions tool for comparative cohort studies, and the Cochrane Risk of Bias 2 tool for randomized controlled studies. For the Risk of Bias in Nonrandomized Studies of Interventions (ROBINS-I) tool^16^, risk of bias judgement per study is noted as low risk when all domains are judged as low risk of bias, moderate risk when one domain is judged as moderate risk of bias, serious risk when one domain is judged as serious risk of bias, or critical risk of bias when one domain is judged as critical risk of bias. For the Risk of Bias in the Cochrane Risk of Bias 2 tool, risk of bias judgement per study is noted as low risk when all domains are judged as low risk of bias, some concerns when one or more domains are judged as some concerns, or high risk when at least one domain is judged as high risk of bias, or when multiple domains are judged as some concerns. Risk of bias for non-comparative cohort studies was regarded as high risk of bias.

### Outcomes of interest

The primary outcomes were the seroconversion rate following the uncomplete, complete, or booster dose of COVID-19 vaccines in patients living with HIV. The secondary outcomes were the risk ratios for seroconversion following the uncomplete, complete, or booster dose of COVID-19 vaccines between HIV-infected and non-HIV-infected patients.

### Statistical analysis

All the analyses were performed and visualized with R statistic software (3.6.3). The principal summary measures used were pooled rate and risk ratio with 95% confidential interval (CI) of seroconversion following COVID-19 vaccination. χ^2^ test and I^2^ statistic were performed to evaluate the statistical heterogeneity of the results in the included studies. We considered heterogeneity to be significant when the P value by χ^2^ test was < 0.1 or the I^2^ statistic was ≥ 50%. The pooled seroconversion rate was assessed using the Freeman-Tukey double arcsine method. The pooled risk ratios were combined by the Mantel–Haenszel approach. Random-effects model was preferentially used as the primary approach to pool results across studies due to underlying clinical heterogeneity (eg, basic characteristics of the patients, COVID-19 history, adjustment for confounders).

Subgroup analyses were conducted following the complete dose of COVID-19 vaccines according to year of publication (2021 vs. 2022), study location (Europe vs. North America vs. South Africa vs. Asia vs. South America), study design (retrospective vs. prospective), source of data (multi-center vs. single-center), sample size (< 100 vs. ≥ 100), follow-up duration (< 2 months vs. ≥ 2 months), adjustment (yes vs. no), antiretroviral therapy (yes vs. not available), COVID-19 history (might be enrolled vs. none), and vaccine type (mRNA vs. adenovirus vs. inactivated vs. other vaccines). Meta-regression analyses were conducted to explore the potential effect of these parameters on the outcomes. The regression coefficient was calculated to describe the change of outcomes with explanatory variables (potential effect modifiers). Sensitivity analyses were conducted where the outcomes were recalculated by omitting one study at a time. Publication bias was evaluated by examining funnel plots (≥ 10 included studies) in combination with Egger’s test. If publication bias existed (funnel plot asymmetry, or Egger’s test P < 0.1), trim-and-fill analyses were performed to adjust for publication bias and further evaluate the stability of the pooled results. P value < 0.05 was considered statistically significant.

## Result

### Study selection, characteristics and quality assessment

**Figure 1:**
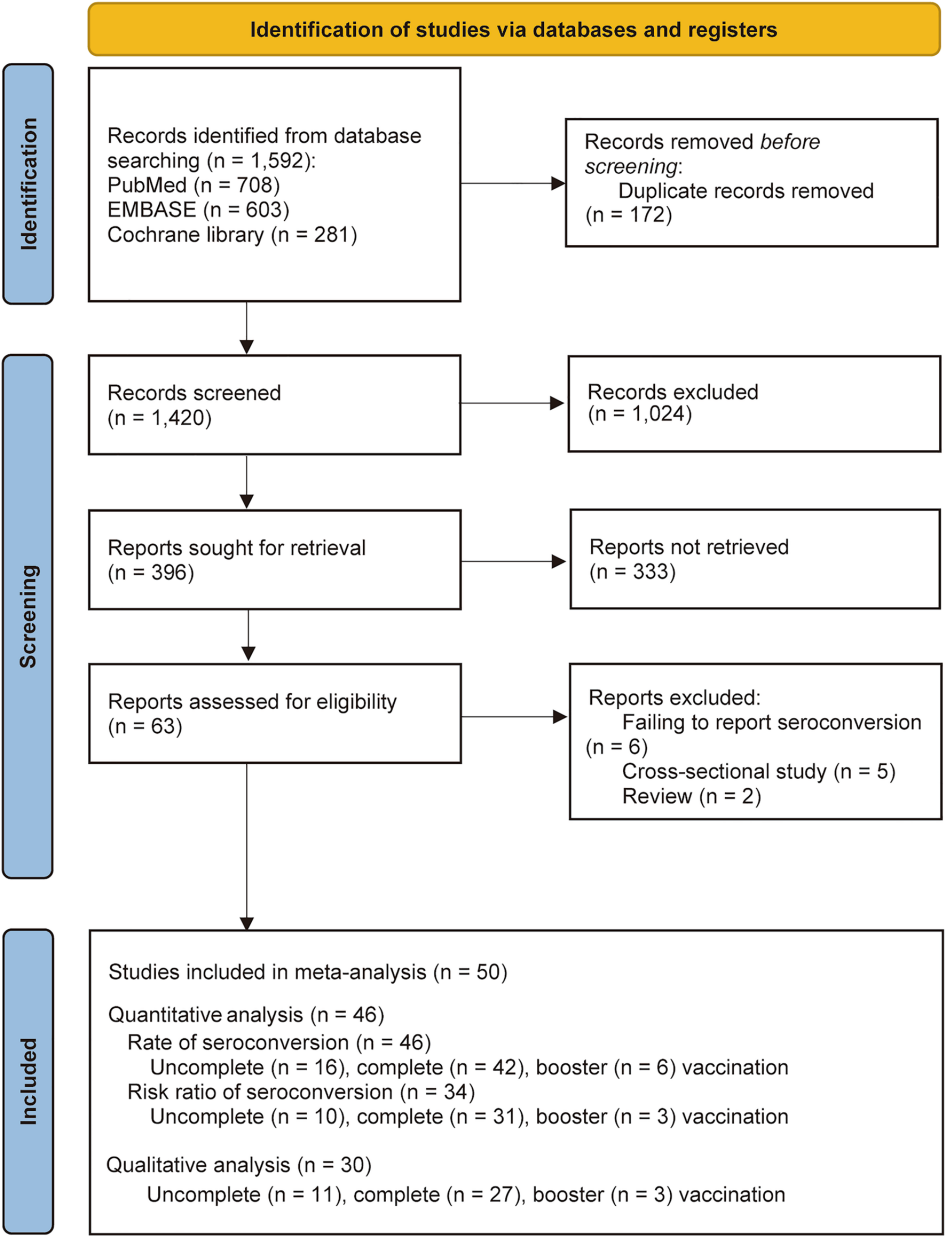
Flowcharts illustrating the article selection process.

We identified 1592 citations through the literature search, excluded 1357 after initial title and abstract screening, and assessed the full text of 63 studies for eligibility. Another 13 studies were further removed for failing to report seroconversion (n = 6), cross-sectional studies (n = 5), reviews (n = 2) (File S1). Finally, 50 studies with a total of 7160 patients living with HIV were included in our meta-analysis^11,12,17–64^, 30 studies were included for qualitative analysis of serological antibody titers (Supplementary Tables S3–S5); 46 studies^11,12,17–25,27–32,34–39,41–56,58–64^ were included for quantitative analysis of pooled seroconversion rate; 34 studies^11,12,17–20,23–25,27,31,35,36,38,39,41–46,48–51,53,55,56,59–64^ were used for quantitative analysis of pooled risk ratios for seroconversion following the uncomplete, complete, or booster dose of COVID-19 vaccines between patients living with HIV and HIV-negative vaccine counterparts (Fig. 1).

The main characteristics and clinical outcomes of the studies for quantitative analysis were summarized in Table 1 and Supplementary Table S2. The included studies were published between 2021 and 2022. Of these studies, 21 were from Europe, 11 from Asia, 9 from North America, 3 from South Africa and 2 from South America. The studies comprised 31 prospective studies and 15 retrospective studies. 19 studies were multicenter and 27 were single-center. The number of patients living with HIV in 18 studies was above 100; the follow-up duration in 15 studies was more than 2 months; only 11 studies had adjusted for potential confounders; the patients living with HIV in 40 studies received antiretroviral therapy; the patients living with HIV in 35 studies were not infected with COVID-19 prior to vaccination. In terms of vaccination type, mRNA vaccines were used in 26 studies; adenovirus vaccines were used in 3 studies; inactivated vaccines were used in 10 studies; and another 7 studies involved two or more vaccines or other types of vaccines. Supplementary Table S2 presents demographic characteristics, immunoassay and threshold for positive response. Supplementary Table S6 shows the detailed risk of bias for each study, and most of studies were regarded as critical or high risk of bias.

**Table 1:** Characteristics of included studies.

Source	Country	Design	Data source	Group	n. populations	Vaccine type	Antiretroviral therapy	Covid_19 history	Duration of follow-up (days)	Adjustments
Bergman 2021	Sweden	Pro	Karolinska University Hospital	PLWH	79	BNT162b2	Not available	None	Complete: 14	age (partially)
				HC	78	BNT162b2	–			
Frater 2021	UK	Pro	Imperial College NHS Trust and Guy’s and St Thomas’NHS Foundation Trust	PLWH	52	ChAdOx1 nCoV-19 (AZD1222)	All receiving ART	None	Uncomplete: 28 Complete: 28	age, ethnicity and dosing strategy
				HC	48	ChAdOx1 nCoV-19 (AZD1222)	–			
Levy 2021	Israel	Pro	Sheba Medical Centre	PLWH	135	BNT162b2	All receiving ART	None	Complete: 18	–
				HC	201	BNT162b2	–			
Madhi 2021	South Africa	RCT	Seven South African locations	PLWH	36	ChAdOx1 nCoV-19 (AZD1222)	All receiving ART	None	Uncomplete: 14 Complete: 14	RCT
				HC	15	ChAdOx1 nCoV-19 (AZD1222)	–			
Rahav 2021	Israel	Pro	Sheba Medical Center	PLWH	156	BNT162b2	All receiving ART	None	Complete: 19	–
				HC	272	BNT162b2	–			
Ruddy 2021	USA	Pro	National HIV/AIDS organizations	PLWH	12	BNT162b2 (50%) or mRNA-1273 (50%)	All receiving ART	None	Uncomplete: 21	–
Woldemeskel 2021	USA	Retro	The Johns Hopkins Center for AIDS Research	PLWH	12	BNT162b2	All receiving ART	None	Complete: 13	–
				HC	17	BNT162b2 (96%) or mRNA-1273 (4%)	–			
Aledo 2022	Spain	Retro	University Hospital of A Coruña	PLWH	100	BNT162b2 (10%) or mRNA-1273 (90%)	All receiving ART	None	Complete: 28	–
Anais 2022	Spain	Pro	Three university hospitals in Southern Spain	PLWH	385	mRNA vaccines (79%) (BNT162b2 or mRNA-1273) or Adenovirus vaccines (21%) (ChAdOx1 nCoV-19 or Ad26.COV2.S)	All receiving ART	None	Complete: 42	–
Antinori 2022	Italy	Pro	National Institute for Infectious Diseases Lazzaro Spallanzani	PLWH	153	BNT162b2 (57.2%) or mRNA-1273 (42.8%)	All receiving ART	None	Complete: 30	–
				HC	73	BNT162b2	–			
Ao 2022	China	Pro	People’s Hospital of Tongliang District	PLWH	30	BBIBP-CorV (24.5%), Corona Vac (48.2%) or BBIBP-CorV + Corona Vac (27.3%)	All receiving ART	None	Complete: 180	–
				HC	27	BBIBP-CorV (44.2%), Corona Vac (50.8%) or BBIBP-CorV + Corona Vac (5%)	–			
Balcells 2022	Chile	Pro	Red de Salud UC-CHRISTUS and collaborating centers	PLWH	55	CoronaVac	All receiving ART	None	Complete: 70	–
				HC	65	CoronaVac	–			
Brumme 2022	Canada	Retro	Three HIV care clinics in Vancouver	PLWH	98	BNT162b2 or mRNA-1273 or ChAdOx1	All receiving ART	None	Uncomplete: 30 Complete: 30	age,chronic health conditions
				HC	151	BNT162b2 or mRNA-1273 or ChAdOx1	–			
Chan 2022	China	Pro	Two major HIV specialist clinics in Hong Kong	PLWH	122	CoronaVac	All receiving ART	None	Complete: 48 Boost: 33	–
Cossu 2022	Italy	Retro	HIV clinical center in Milan	PLWH	53	BNT162b2	All receiving ART	Might be enrolled	Complete: 189	–
				HC	34	BNT162b2	–			
Feng 2022	China	Pro	Hubei Provincial Center for Disease Control and Prevention	PLWH	42	BBIBP-CorV	All receiving ART	None	Uncomplete: 28 Complete: 28	–
				HC	28	BBIBP-CorV	–			
Gianserra 2022	Italy	Pro	HIV/AIDS Unit of the San Gallicano Dermatological Institute	PLWH	42	BNT162b2	All receiving ART	None	Complete: 166 Boost: 28	–
Haidar 2022	USA	Pro	Unive University of Pittsburgh Medical Center Health System	PLWH	94	BNT162b2(67.0%), mRNA-1273 (30.9%), or Adenovirus (2.1%)	All receiving ART	None	Complete: 85.5	–
				HC	172	BNT162b2 (55.8%), mRNA-1273 (42.4%) or Adenovirus (1.7%)	–			
Han 2022	China	Retro	Beijing Ditan Hospital	PLWH	10	CoronaVac or Sinopharm	All receiving ART	None	Complete:28	age, sex, and interval length
				HC	18	CoronaVac or Sinopharm				
Hassold 2022	France	Retro	Department of Infectious Diseases of Hospital Avicenne	PLWH	105	BNT162b2(75%), mRNA-1273(8.5%) or ChAdOx1-nCoV19(16.5%)	86.7% receiving ART	None	Complete: 73	–
Heftdal 2022	Denmark	Pro	Copenhagen University Hospital	PLWH	269	BNT162b2	99.6% receiving ART	None	Uncomplete: 21 Complete: 60	age
				HC	538	BNT162b2	–			
Hensley 2022	Netherlands	Pro	22 HIV treatment centres	PLWH	1154	BNT162b2(76.6%), mRNA-1273(8.7%), ChAdOx1-S (13.0%) or Ad26.COV2.S (1.7%)	99.0% receiving ART	None	Complete: 35	–
				HC	440	BNT162b2(21.4%), mRNA-1273(56.1%), ChAdOx1-S (5.9%) or Ad26.COV2.S(16.6%)	–			
Khan 2022	South African	Pro	Biomedical Research of University of KwaZulu–Natal	PLWH	26	Ad26.CoV2.S	ART	Might be enrolled	Complete: 62.5	–
				HC	73	Ad26.CoV2.S	–	Might be enrolled		
Lapointe 2022	Canada	Retro	University of British Columbia/Providence Health Care	PLWH	56	BNT162b2 or mRNA-1273	All receiving ART	None	Boost: 30	age, chronic health conditions
				HC	107	BNT162b2 or mRNA-1273	–			
Lombardi 2022	Italy	Pro	The Infectious Diseases Unit of the IRCCS Ospedale Maggiore Policlinico in Milan	PLWH	71	mRNA-1273	All receiving ART	Might be enrolled	Complete: 28	–
				HC	10	mRNA-1273	–	Might be enrolled		
Loubet 2022	France	Pro	36 centres in France	PLWH	1111	BNT162b2 or mRNA-1273	Not available	None	Complete: 30 Boost: 30	–
				HC	873	BNT162b2 or mRNA-1273	–			
Lv 2022	China	Retro	Malipo Country People’s Hospital	PLWH	24	BBIBP-CorV or CoronaVac	Not available	None	Complete: 40	–
				HC	24	BBIBP-CorV or CoronaVac	–			
Madhi 2022	South Africa	RCT	16 academic and private clinic research sites	PLWH	101	NVX-CoV2373	All receiving ART	Might be enrolled	Uncomplete: 21 Complete: 14	RCT
				HC	1899	NVX-CoV2373	–	Might be enrolled		
Milano 2022	Italy	Pro	University of Bari	PLWH	694	BNT162b2	All receiving ART (except one long‐term non-progressor);	None	Uncomplete: 21 Complete: 90	–
Nault 2022	Canada	Retro	HIV clinics in Montreal	PLWH	106	mRNA-1273	All receiving ART	Might be enrolled	Uncomplete: 28	–
				HC	20	BNT162b2	–	Might be enrolled		
Netto 2022	Brazil	Pro	University of Sao Paulo HIV/AIDS outpatient clinic	PLWH	211	CoronaVac	99.5% receiving ART	None	Uncomplete: 28 Complete: 42	–
				HC	289	CoronaVac	–			
Oyaert 2022	Belgium	Pro	Ghent University Hospital	PLWH	27	BNT162b2	ART	Might be enrolled	Uncomplete: 24.5 Complete: 90	–
				HC	54	BNT162b2	–	Might be enrolled		
Polvere 2022	Italy	Retro	Azienda Ospedaliera Universitaria Senese	PLWH	84	BNT162b2(48.8%) or mRNA-1273(51.2%)	All receiving ART	None	Complete: 150	–
				HC	79	BNT162b2(87.3%) or mRNA-1273(12.7%)	–			
Portillo 2022	Switzerland	Retro	Geneva University Hospital	PLWH	129	BNT162b2(40.5%) or mRNA-1273(59.5%)	ART	Might be enrolled	Uncomplete: 28 Complete: 150	–
				HC	49	mRNA-1273	–			
Pourcher 2022	France	Pro	The infectious disease departments of the AP-HP Sorbonne Universit	PLWH	90	BNT162b2	All receiving ART	Might be enrolled	Uncomplete: 28 Complete: 30	–
Ruddy 2022	USA	Pro	National HIV/AIDS organizations	PLWH	14	BNT162b2 (36%) or mRNA-1273(64%)	All receiving ART	None	Uncomplete: 21 Complete: 29	–
Schmidt 2022	Germany	Pro	Erlangen HIV cohort	PLWH	50	BNT162b2	All receiving ART	None	Complete: 37	–
				HC	57	BNT162b2	-			
Speich 2022	Switzerland	RCT	University Hospital Basel, University Hospital Bern and University Hospital Zurich	PLWH	338	BNT162b2(50%) or mRNA-1273(50%)	Not available	Might be enrolled	Complete: 56	RCT
Spinelli 2022	USA	Retro	A large outpatient HIV clinic	PLWH	100	BNT162b2 (75%) or mRNA-1273 (25%)	Not available	None	Complete: 35	care for chronic medical conditions on days since completion of 2nd vaccination (minimum 10), sex, age ± 5 years, and the type of mRNA vaccine received
				HC	100	BNT162b2 (75%) or mRNA-1273 (25%)	–			
Tan 2022	China	Pro	Zhongnan Hospital of Wuhan University	PLWH	41	Sinopharm	All receiving ART	None	Boost: 14	–
				HC	18	Sinopharm	–			
Tuan 2022	USA	Retro	Two HIV clinics of the Yale New Haven Health System	PLWH	78	BNT162b2	All receiving ART	None	Uncomplete: 21 Complete: 17.5	–
Vergori 2022	Italy	Retro	Infectious Diseases Lazzaro Spallanzani in Rome	PLWH	106	BNT162b2 or mRNA-1273	All receiving ART	None	Complete: 156 Boost: 14	–
				HC	28	BNT162b2 or mRNA-1273	–			
Wong 2022	China	Pro	The Integrated Treatment Centre or Princess Margaret Hospital HIV Service	PLWH	19	CoronaVac (31%) or Comirnaty (69%)	Not available	None	Uncomplete: 24.5 Complete: 180	age, sex, CD4 + cell count, and suppressed viral load (SVL) at the timepoint nearest to vaccination
				HC	35	CoronaVac (28.5%) or Comirnaty (71.5%)	–			
Xu 2022	Sweden	Pro	Karolinska University Hospital	PLWH	79	BNT162b2	All receiving ART	None	Complete: 14	–
				HC	82	BNT162b2	–			
Zeng 2022	China	Retro	The Third People’s Hospital of Shenzhen	PLWH	99	BBIBP-CorV (49.2%) or CoronaVac (50.8%)	95.5% receiving ART	None	Complete: 180	–
				HC	83	BBIBP-CorV (50%) or CoronaVac (50%)	–			
Zou 2022	China	Pro	Wuchang district of Wuhan city	PLWH	35	Sinopharm WIBP-CorV	All receiving ART	None	Complete: 42	–
				HC	38	Sinopharm WIBP-CorV	–			

PLWH, people living with HIV; HC, healthy control; UK, United Kingdom; USA, United States of America; IQR, interquartile range; Pro, Prospective study; Retro, retrospective study; RCT, randomized controlled trial; ART, antiretroviral therapy; AIDS, Acquired Immune Deficiency Syndrome.

### Seroconversion rate after uncomplete, complete, and booster vaccination

**Figure 2:**
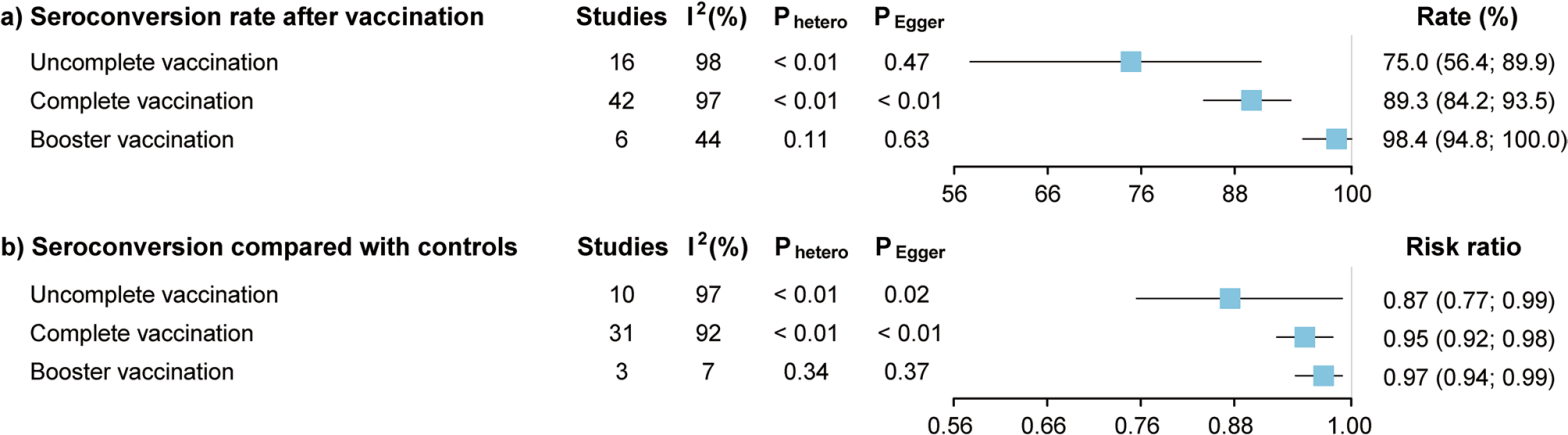
The pooled rate (**a**) and risk ratio (**b**) of seroconversion after uncomplete, complete, or booster vaccination in patients with living HIV.

16 studies, 42 studies, and 6 studies evaluated the seroconversion rate of patients living with HIV after uncomplete, complete, and booster vaccination, respectively. As shown in Fig. 2a, the seroconversion rate was 75.0% (95% CI 56.4% to 89.9%) after uncomplete vaccination, 89.3% (95% CI 84.2% to 93.5%) after complete vaccination, and 98.4% (95% CI 94.8% to 100%) after booster vaccination. Significant heterogeneity was seen for the pooled seroconversion rate after uncomplete vaccination (I^2^ > 50%, P < 0.10) (Supplementary Fig. S1a). The funnel plot and Egger’s test (P = 0.47) did not detect the existence of publication bias in these studies (Supplementary Fig. S1b). The sensitivity analysis performed by using the “leave-one-out” did not markedly change our results (Supplementary Fig. S1c). Also, there is significant heterogeneity for the pooled seroconversion rate after complete vaccination (I^2^ > 50%, P < 0.10) (Supplementary Fig. S2). The funnel plot and Egger’s test (P < 0.01) suggested the existence of publication bias in these studies (Supplementary Fig. S3a). After 10 studies were filled, the funnel plot showed the relative symmetry (Supplementary Fig. S3b), and Egger’s test showed no evidence of significant publication bias (P = 0.49). The pooled seroconversion rate turned to be 96.6% (95% CI 92.6% to 99.2%) after complete vaccination. The sensitivity analysis did not significantly change our results (Supplementary Fig. S3c). As for the pooled seroconversion rate after booster vaccination, moderate heterogeneity was observed (I^2^ = 44%, P = 0.11) (Supplementary Fig. S4a), and the funnel plot showed the relative symmetry (Supplementary Fig. S4b), and Egger’s test showed no evidence of significant publication bias (P = 0.63). The results were stable after sensitivity analysis (Supplementary Fig. S4c).

### Seroconversion compared with controls after uncomplete, complete, and booster vaccination

10 studies, 31 studies, and 3 studies compared the seroconversion with HIV-negative vaccine counterparts after uncomplete, complete, and booster vaccination. As suggested in Fig. 2b, the risk ratios were 0.87 (95% CI 0.77 to 0.99) after uncomplete vaccination, 0.95 (95% CI 0.92 to 0.98) after complete vaccination, and 0.97 (95% CI 0.94 to 0.99) after booster vaccination. Significant heterogeneity was seen for the pooled risk ratios for seroconversion after uncomplete vaccination (I^2^ > 50%, P < 0.10) (Supplementary Fig. S5a). The funnel plot and Egger’s test (P < 0.01) suggested the existence of publication bias in these studies (Supplementary Fig. S5b). After 5 studies were filled, the funnel plot showed relative symmetry (Supplementary Fig. S5c), and Egger’s test showed no evidence of significant publication bias (P = 0.89). The pooled risk ratios for seroconversion changed to 1.01 (95% CI 0.95 to 1.09) after uncomplete vaccination. The sensitivity analysis performed by using the “leave-one-out” did not markedly change our results except omitting Feng’s, Netto’s or Wong’s study (Supplementary Fig. S5d). Moreover, there is significant heterogeneity for the pooled seroconversion rate after complete vaccination (I^2^ > 50%, P < 0.10) (Supplementary Fig. S6). The funnel plot and Egger’s test (P < 0.01) suggested the existence of publication bias in these studies (Supplementary Fig. S7a). After 13 studies were filled, the funnel plot showed relative symmetry (Supplementary Fig. S7b), and Egger’s test showed no evidence of significant publication bias (P = 0.78). The pooled seroconversion rate turned to be 1.00 (95% CI 0.98 to 1.03) after complete vaccination. The sensitivity analysis did not significantly change our results (Supplementary Fig. S7c). Besides, there was minimal heterogeneity for seroconversion after booster vaccination (I^2^ = 7%, P = 0.34) (Supplementary Fig. S8a), and the funnel plot showed relative symmetry (Supplementary Fig. S8b), and Egger’s test showed no evidence of significant publication bias (P = 0.37). The results were stable after sensitivity analysis except omitting Vergori’s study (Supplementary Fig. S8c).

### Meta-regression and subgroup analysis for seroconversion rate after complete vaccination

**Figure 3:**
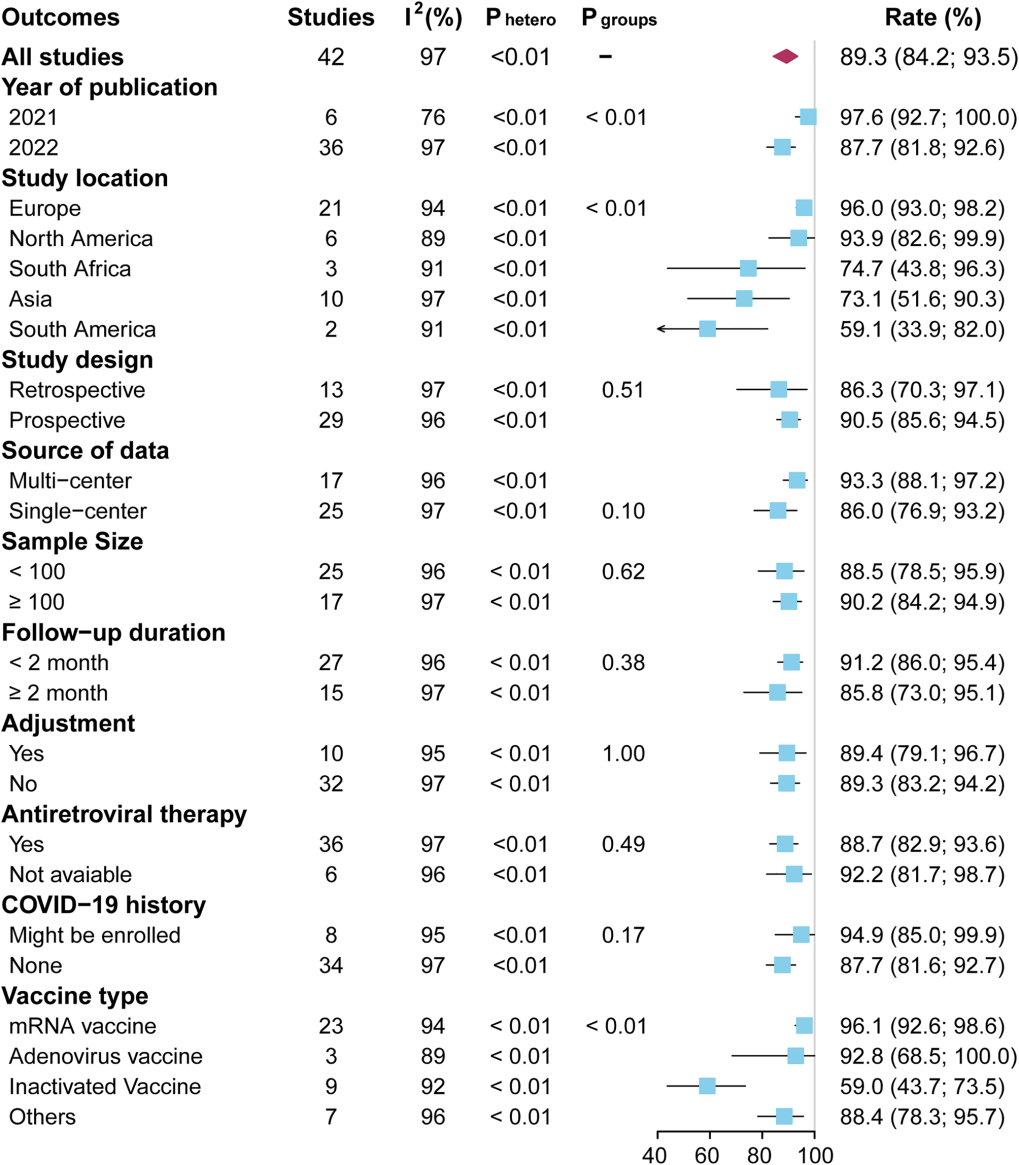
Subgroup analyses of the pooled seroconversion rate after complete vaccination in patients with living HIV.

To examine whether the observed heterogeneity could be contributed by possible moderators for the pooled seroconversion rate after complete vaccination, univariate meta-regression was performed and suggested that study location and vaccine type were possible significant moderators (Supplementary Table S7). Subgroup analyses were further performed to evaluate the potential mediators for the pooled seroconversion rate after complete vaccination (Fig. 3, Supplementary Figs. S9–S18). Subgroup analysis according to year of publication demonstrated that the rate was lower in studies published in 2022, compared with studies published in 2021 (87.7% vs. 97.6%, P < 0.01). Subgroup analysis on basis of study location suggested that the rate was lowest in South America (59.1%), compared with Asia (73.1%), South Africa (74.7%), North America (93.9%), Europe (96.0%) (P < 0.01). Subgroup analysis stratified by vaccine type showed that the rate was lowest with inactivated vaccine (59%), compared with adenovirus vaccine (92.8%), mRNA vaccine (96.1%) or other vaccines (88.4%) (P < 0.01). There was no significant heterogeneity among all subgroup comparisons (all P > 0.05) when subgroup analyses were based on study design, source of data, sample size, follow-up duration, adjustment, antiretroviral therapy, or COVID-19 history.

### Meta-regression and subgroup analysis for seroconversion compared with controls after complete vaccination

**Figure 4:**
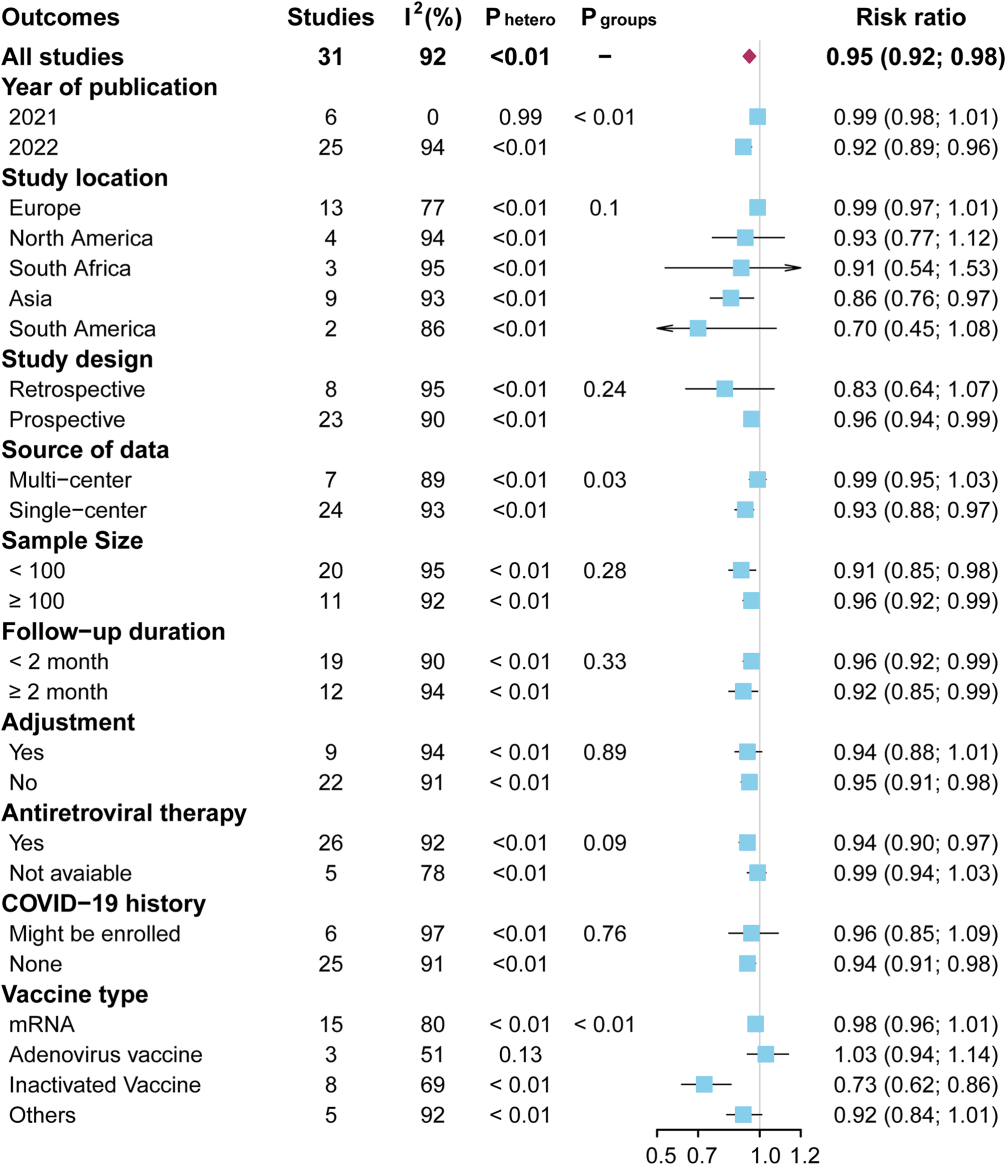
Subgroup analyses of the pooled risk ratio of seroconversion after complete vaccination between patients with living HIV and controls.

Univariate meta-regression was further performed to explore the origin of heterogeneity for seroconversion compared with controls after complete vaccination, and results showed that study location and vaccine type were also possible significant moderators (Supplementary Table S8). Subgroup analyses were further performed to evaluate the potential mediators for the pooled seroconversion compared with controls after complete vaccination (Fig. 4, Supplementary Figs. S19–S28). Subgroup analysis according to year of publication demonstrated that the risk ratio was lower in studies published in 2022, compared with studies published in 2021 (0.92 vs. 0.99, P < 0.01). Subgroup analysis on basis of source data suggested that the risk ratio was lower in single-center studies (0.93), compared with multi-center studies (0.99) (P = 0.03). Subgroup analysis stratified by vaccine type showed that the risk ratio was lowest with inactivated vaccine (0.73), compared with mRNA vaccine (0.98), adenovirus vaccine (1.03), or other vaccines (0.92) (P < 0.01). There was no significant heterogeneity among all subgroup comparisons (all P > 0.05) when subgroup analyses were based on study location, study design, sample size, follow-up duration, adjustment, antiretroviral therapy, or COVID-19 history.

### Grading the quality of evidence

According to the GRADE approach, the quality of evidence was very low for seroconversion rate after uncomplete or complete vaccination, and the quality of evidence was low for overall seroconversion rate after booster vaccination (Supplementary Table S9a). The quality of evidence was low for seroconversion compared with controls after uncomplete or complete vaccination, and the quality of evidence was moderate for seroconversion compared with controls after booster vaccination (Supplementary Table S9b). Supplementary Table S9 provided the detailed criteria to down- or up- grade the level certainty.

## Discussion

COVID-19 pandemic has ravaged across the globe, claiming the lives of more than 6 million people^65^. COVID-19 vaccines have been found to be the main measure of reducing the severity and mortality of COVID-19 patients^5,6^. Increasing studies indicated impaired serological response following vaccination in immunocompromised patients with cancer^66–68^, immune-mediated inflammatory disorders^69–71^, or organ transplant^72–74^. However, data are scarce on COVID-19 vaccination responses in patients living with HIV.

In this meta-analysis, we analyzed 50 studies with a total of 7160 patients living with HIV. We demonstrated that only 75.0% patients living with HIV achieved a seroconversion after uncomplete vaccination, which improved to 89.3% after complete vaccination, and 98.4% after booster vaccination. The seroconversion rates were significantly lower compared to controls at all the stages, while the risk ratios for uncomplete, complete, and booster vaccination were 0.87, 0.95, and 0.97, respectively, suggesting the urgent need for booster vaccination in patients living with HIV.

In the meta-regression and subgroup analysis, we found that year of publication, study location and vaccine type were possible significant moderators for the pooled rate and risk ratio for seroconversion after complete vaccination. As for year of publication, the seroconversion rate was lower in studies published in 2022, compared with studies published in 2021. A possible explanation is that virus variation weakens the effectiveness of the vaccine over time^75–77^. Regarding study location, the seroconversion rate was the lowest in South America, followed by Asia, South Africa, North America, and Europe. These location-specific differences were partly because of different vaccine types in these regions^78,79^. Moreover, Liu et al. previously predicted SARS-CoV-2 has different peptide-HLA hits for MHC class I and MHC class II peptides in white, black and Asian ancestry^80^, which could cause the difference in these regions. It is also worth noting that vaccine types affected the seroconversion in patients living with HIV, and the seroconversion rate was lowest with inactivated vaccine, followed by adenovirus vaccine, and mRNA vaccine. Kwok et al. and Peng et al. previously demonstrated that compared with mRNA vaccine, the antibody level of inactivated CoronaVac-vaccinees wane quickly and patients after the vaccine face a higher risk of breakthrough infection^81,82^. Besides, Alhinai et al. performed longitudinal analyses of publicly accessible epidemiological, clinical, virological, vaccine-related, and other public health data from 41 eligible countries, and found that the real-world effectiveness of inactivated virus vaccines might be inferior to mRNA and/or adenovirus-vectored vaccines^83^. Our results further validated previous findings, and provided solid evidence through comprehensively analyzing all the published papers. However, the subgroup differences we found highlight the need for high quality studies on these differences, specifically the improvement in the design of studies, greater geographical representation and comparison of vaccine types.

Admittedly, our study has several limitations. First, notable heterogeneity was found in some comparisons, which may be attributed to various immunoassay kits, threshold for seroconversion, and immune status at the time of COVID-19 vaccination in patients living with HIV^29,31,84^. However, sensitivity analysis, subgroup analysis and trim-and-fill analysis were used for meta-analysis, suggesting the stability of the results. Second, significant publication bias was observed in some comparisons, partly because most of studies enrolled were on mRNA vaccines, which could cause some bias in the results. Thirdly, here we failed to explore the effect of CD4 T cell absolute counts on the seroconversion of COVID-19 vaccines in HIV patients. This gap was filled by our other study showing that CD4 T cell count is positively correlated with seroconversion among COVID-19 vaccinated patients with HIV^85^. Finally, the rate of seroconversion was pooled after the uncomplete, complete, and booster vaccination. However, seroconversion rate is just an indicator of vaccine immune response and surrogate endpoints for the vaccine's impact on infection rates and the severity of COVID-19^86–88^. Data on clinical efficacy endpoints, such as COVID-19 infection rates in vaccinated patients living with HIV, are still lacking^89^.

## Conclusion

Our meta-analysis summarized the pooled seroconversion rate and the pooled risk ratios following the uncomplete, complete, or booster dose of COVID-19 vaccines in patients living with HIV. We concluded that vaccine doses were associated with consistently improved seroconversion rates and risk ratios in patients living with HIV. Our study provides solid evidence that booster vaccination is necessary for patients living with HIV.

## Supplementary Information

Supplementary Information.

## Data Availability

The data that support the findings of this study are available from the corresponding author upon reasonable request.

## References

[1] Geretti AM (2021). Outcomes of coronavirus disease 2019 (COVID-19) related hospitalization among people with human immunodeficiency virus (HIV) in the ISARIC World Health Organization (WHO) Clinical Characterization Protocol (UK): A prospective observational study. Clin. Infect. Dis..

[2] Western Cape Department of Health in collaboration with the National Institute for Communicable Diseases, S. A. (2021). Risk factors for coronavirus disease 2019 (COVID-19) death in a population cohort study from the Western Cape Province, South Africa. Clin. Infect. Dis..

[3] Tesoriero JM (2021). COVID-19 outcomes among persons living with or without diagnosed HIV infection in New York State. JAMA Netw. Open.

[4] Bhaskaran K (2021). HIV infection and COVID-19 death: A population-based cohort analysis of UK primary care data and linked national death registrations within the OpenSAFELY platform. Lancet HIV.

[5] Polack FP (2020). Safety and efficacy of the BNT162b2 mRNA Covid-19 vaccine. N. Engl. J. Med..

[6] Dagan N (2021). BNT162b2 mRNA Covid-19 vaccine in a nationwide mass vaccination setting. N. Engl. J. Med..

[7] Xu H (2022). Effectiveness of inactivated COVID-19 vaccines against mild disease, pneumonia, and severe disease among persons infected with SARS-CoV-2 Omicron variant: Real-world study in Jilin Province, China. Emerg. Microb. Infect..

[8] Mallory RM (2022). Safety and immunogenicity following a homologous booster dose of a SARS-CoV-2 recombinant spike protein vaccine (NVX-CoV2373): A secondary analysis of a randomised, placebo-controlled, phase 2 trial. Lancet Infect. Dis..

[9] Catherine FX, Piroth L (2017). Hepatitis B virus vaccination in HIV-infected people: A review. Hum. Vaccin. Immunother..

[10] Pallikkuth S (2018). Impact of aging and HIV infection on serologic response to seasonal influenza vaccination. AIDS.

[11] Madhi SA (2021). Safety and immunogenicity of the ChAdOx1 nCoV-19 (AZD1222) vaccine against SARS-CoV-2 in people living with and without HIV in South Africa: An interim analysis of a randomised, double-blind, placebo-controlled, phase 1B/2A trial. Lancet HIV.

[12] Bergman P (2021). Safety and efficacy of the mRNA BNT162b2 vaccine against SARS-CoV-2 in five groups of immunocompromised patients and healthy controls in a prospective open-label clinical trial. EBioMedicine.

[13] Lee A (2022). Efficacy of covid-19 vaccines in immunocompromised patients: systematic review and meta-analysis. BMJ.

[14] Page MJ (2021). The PRISMA 2020 statement: an updated guideline for reporting systematic reviews. BMJ.

[15] Dekkers OM, Egger M, Altman DG, Vandenbroucke JP (2012). Distinguishing case series from cohort studies. Ann. Intern. Med..

[16] Sterne JA (2016). ROBINS-I: a tool for assessing risk of bias in non-randomised studies of interventions. BMJ.

[17] Frater J (2021). Safety and immunogenicity of the ChAdOx1 nCoV-19 (AZD1222) vaccine against SARS-CoV-2 in HIV infection: A single-arm substudy of a phase 2/3 clinical trial. Lancet HIV.

[18] Levy I (2021). Immunogenicity and safety of the BNT162b2 mRNA COVID-19 vaccine in people living with HIV-1. Clin. Microbiol. Infect..

[19] Portillo V (2021). Impact on HIV-1 RNA levels and antibody responses following SARS-CoV-2 vaccination in HIV-infected individuals. Front. Immunol..

[20] Rahav G (2021). BNT162b2 mRNA COVID-19 vaccination in immunocompromised patients: A prospective cohort study. EClinicalMedicine.

[21] Ruddy JA (2021). Safety and antibody response to two-dose SARS-CoV-2 messenger RNA vaccination in persons with HIV. AIDS.

[22] Ruddy JA (2021). Safety and antibody response to the first dose of severe acute respiratory syndrome coronavirus 2 messenger RNA vaccine in persons with HIV. AIDS.

[23] Antinori A (2022). Humoral and cellular immune response elicited by mRNA vaccination against severe acute respiratory syndrome coronavirus 2 (SARS-CoV-2) in people living with human immunodeficiency virus receiving antiretroviral therapy based on current CD4 T-lymphocyte count. Clin. Infect. Dis..

[24] Ao L (2022). Safety and immunogenicity of inactivated SARS-CoV-2 vaccines in people living with HIV. Emerg. Microb. Infect..

[25] Balcells ME (2022). Reduced immune response to inactivated severe acute respiratory syndrome coronavirus 2 vaccine in a cohort of immunocompromised patients in Chile. Clin. Infect. Dis..

[26] Bessen C (2022). Impact of SARS-CoV-2 vaccination on systemic immune responses in people living with HIV. Front. Immunol..

[27] Brumme ZL (2022). Humoral immune responses to COVID-19 vaccination in people living with HIV receiving suppressive antiretroviral therapy. npj Vaccines.

[28] Chan DPC, Wong NS, Wong BCK, Chan JMC, Lee SS (2022). Three-dose primary series of inactivated COVID-19 vaccine for persons living with HIV, Hong Kong. Emerg. Infect. Dis..

[29] Corma-Gómez A (2022). Severe immunosuppression is related to poorer immunogenicity to SARS-CoV-2 vaccines among people living with HIV. Clin. Microbiol. Infect..

[30] Cossu MV (2022). Does the co-morbidity burden contribute to suboptimal immunological responses to COVID-19 vaccination in people living with HIV?. J. Infect. Dis..

[31] Feng Y (2022). Immunogenicity of an inactivated SARS-CoV-2 vaccine in people living with HIV-1: A non-randomized cohort study. EClinicalMedicine.

[32] Gianserra L (2022). Immunogenicity and safety of BNT162b2 homologous booster vaccination in people living with HIV under effective cART. Vaccines.

[33] Gidari A (2022). BNT162b2 elicited an efficient humoral response against different strains of SARS-CoV-2 in people living with HIV. Curr. HIV Res..

[34] Gonzalez de Aledo M (2022). Safety and immunogenicity of SARS-CoV-2 vaccines in people with HIV. AIDS.

[35] Haidar G (2022). Prospective evaluation of coronavirus disease 2019 (COVID-19) vaccine responses across a broad spectrum of immunocompromising conditions: The COVID-19 vaccination in the immunocompromised study (COVICS). Clin. Infect. Dis..

[36] Han X (2022). Safety and immunogenicity of inactivated COVID-19 vaccines among people living with HIV in China. Infect. Drug Resist..

[37] Hassold N (2022). Impaired antibody response to COVID-19 vaccination in advanced HIV infection. AIDS.

[38] Heftdal LD (2022). Humoral response to two doses of BNT162b2 vaccination in people with HIV. J. Intern. Med..

[39] Hensley KS (2022). Immunogenicity and reactogenicity of SARS-CoV-2 vaccines in people living with HIV in the Netherlands: A nationwide prospective cohort study. PLoS Med.

[40] Jedicke N (2022). Humoral immune response following prime and boost BNT162b2 vaccination in people living with HIV on antiretroviral therapy. HIV Med..

[41] Khan K (2022). Immunogenicity of severe acute respiratory syndrome coronavirus 2 (SARS-CoV-2) infection and Ad26.CoV2.S vaccination in people living with human immunodeficiency virus (HIV). Clin. Infect. Dis..

[42] Lapointe HR (2022). People with HIV receiving suppressive antiretroviral therapy show typical antibody durability after dual COVID-19 vaccination, and strong third dose responses. J. Infect. Dis..

[43] Lombardi A (2022). Anti-spike antibodies and neutralising antibody activity in people living with HIV vaccinated with COVID-19 mRNA-1273 vaccine: A prospective single-centre cohort study. Lancet Reg. Health. Eur..

[44] Loubet P (2019). One-month humoral response following two or three doses of messenger RNA coronavirus disease 2019 vaccines as primary vaccination in specific populations in France: First results from the Agence Nationale Recherche contre le Sida (ANRS)0001S COV-POPART cohort. Clin. Microbiol. Infect..

[45] Lv Z (2022). Inactivated SARS-CoV-2 vaccines elicit immunogenicity and T-cell responses in people living with HIV. Int. Immunopharmacol..

[46] Madhi SA (2022). Immunogenicity and safety of a SARS-CoV-2 recombinant spike protein nanoparticle vaccine in people living with and without HIV-1 infection: a randomised, controlled, phase 2A/2B trial. Lancet HIV.

[47] Milano E (2022). Immunogenicity and safety of the BNT162b2 COVID-19 mRNA vaccine in PLWH: A monocentric study in Bari, Italy. J. Med. Virol..

[48] Nault L (2022). Covid-19 vaccine immunogenicity in people living with HIV-1. Vaccine.

[49] Netto LC (2022). Safety and immunogenicity of CoronaVac in people living with HIV: a prospective cohort study. Lancet HIV.

[50] Oyaert M (2022). Evaluation of humoral and cellular responses in SARS-CoV-2 mRNA vaccinated immunocompromised patients. Front. Immunol..

[51] Polvere J (2023). B cell response after SARS-CoV-2 mRNA vaccination in people living with HIV. Commun. Med. (Lond).

[52] Pourcher V (2022). High seroconversion rate and SARS-CoV-2 Delta neutralization in people with HIV vaccinated with BNT162b2. AIDS.

[53] Schmidt KG (2022). Characterization of serum and mucosal SARS-CoV-2-antibodies in HIV-1-infected subjects after BNT162b2 mRNA vaccination or SARS-CoV-2 infection. Viruses.

[54] Speich B (2022). Antibody response in immunocompromised patients after the administration of SARS-CoV-2 vaccine BNT162b2 or mRNA-1273: A randomised controlled trial. Clin. Infect. Dis..

[55] Spinelli MA (2022). Differences in post-mRNA vaccination severe acute respiratory syndrome coronavirus 2 (SARS-CoV-2) immunoglobulin G (IgG) concentrations and surrogate virus neutralization test response by human immunodeficiency virus (HIV) status and type of vaccine: A matched case-control observational study. Clin. Infect. Dis..

[56] Tan Y (2022). Early efficacy and safety of the third dose inactivated COVID-19 vaccine among people living with HIV. J. Acquir. Immune Defic. Syndr..

[57] Tau L (2022). SARS-CoV-2 humoral and cellular immune responses of patients with HIV after vaccination with BNT162b2 mRNA COVID-19 vaccine in the Tel-Aviv Medical Center. Open Forum Infect. Dis..

[58] Tuan JJ (2022). Qualitative assessment of anti-SARS-CoV-2 spike protein immunogenicity (QUASI) after COVID-19 vaccination in older people living with HIV. HIV Med..

[59] Vergori A (2022). SARS-CoV-2 omicron variant neutralization after third dose vaccination in PLWH. Viruses.

[60] Woldemeskel BA (2022). The BNT162b2 mRNA vaccine elicits robust humoral and cellular immune responses in people living with human immunodeficiency virus (HIV). Clin. Infect. Dis..

[61] Wong NS (2022). Surrogate neutralization responses following severe acute respiratory syndrome coronavirus 2 vaccination in people with HIV: Comparison between inactivated and mRNA vaccine. AIDS.

[62] Xu X, Vesterbacka J, Aleman S, Nowak P (2022). High seroconversion rate after vaccination with mRNA BNT162b2 vaccine against SARS-CoV-2 among people with HIV - but HIV viremia matters?. AIDS.

[63] Zeng G (2022). IgG antibody responses and immune persistence of two doses of BBIBP-CorV vaccine or CoronaVac vaccine in people living with HIV (PLWH) in Shenzhen, China. Vaccines.

[64] Zou S (2022). Immune response and safety to inactivated COVID-19 vaccine: A comparison between people living with HIV and HIV-naive individuals. AIDS Res. Ther..

[65] World Health Organization,* Coronavirus (COVID-19) Dashboard WHO Coronavirus (COVID-19) Dashboard with Vaccination Data*, https://covid19.who.int/.

[66] Massarweh A (2021). Evaluation of seropositivity following BNT162b2 messenger RNA vaccination for SARS-CoV-2 in patients undergoing treatment for cancer. JAMA Oncol..

[67] Bird S (2021). Response to first vaccination against SARS-CoV-2 in patients with multiple myeloma. Lancet Haematol..

[68] Roeker LE (2021). COVID-19 vaccine efficacy in patients with chronic lymphocytic leukemia. Leukemia.

[69] Reuken PA (2022). T Cell response after SARS-CoV-2 vaccination in immunocompromised patients with inflammatory Bowel disease. J. Crohns Colitis.

[70] Haberman RH (2021). Methotrexate hampers immunogenicity to BNT162b2 mRNA COVID-19 vaccine in immune-mediated inflammatory disease. Ann. Rheum. Dis..

[71] Seyahi E (2021). Antibody response to inactivated COVID-19 vaccine (CoronaVac) in immune-mediated diseases: a controlled study among hospital workers and elderly. Rheumatol. Int..

[72] Grupper A (2021). Kidney transplant recipients vaccinated before transplantation maintain superior humoral response to SARS-CoV-2 vaccine. Clin. Transplant..

[73] Miele M (2021). Impaired anti-SARS-CoV-2 humoral and cellular immune response induced by Pfizer-BioNTech BNT162b2 mRNA vaccine in solid organ transplanted patients. Am. J. Transplant..

[74] Peled Y (2021). BNT162b2 vaccination in heart transplant recipients: Clinical experience and antibody response. J. Heart Lung Transplant..

[75] Li X (2022). Long-term variations and potency of neutralizing antibodies against Omicron subvariants after CoronaVac-inactivated booster: A 7-month follow-up study. J. Med. Virol..

[76] Liang HY (2022). SARS-CoV-2 variants, current vaccines and therapeutic implications for COVID-19. Vaccines.

[77] World Health Organization.* SARS-CoV-2 variants, working definitions and actions taken*, https://www.who.int/activities/tracking-SARS-CoV-2-variants (2022).

[78] Abdelhafiz AS (2022). Sinopharm's BBIBP-CorV vaccine and ChAdOx1 nCoV-19 vaccine are associated with a comparable immune response against SARS-CoV-2. Vaccines.

[79] *France 24 Egypt Plans to Make 1 Billion Sinovac Vaccines a Year*, https://www.france24.com/en/live-news/20210901-egypt-plans-to-make-1-billion-sinovac-vaccines-a-year.

[80] Liu G, Carter B, Gifford DK (2021). Predicted cellular immunity population coverage gaps for SARS-CoV-2 subunit vaccines and their augmentation by compact peptide sets. Cell Syst..

[81] Peng Q (2022). Waning immune responses against SARS-CoV-2 variants of concern among vaccinees in Hong Kong. EBioMedicine.

[82] Kwok SL (2022). Waning antibody levels after COVID-19 vaccination with mRNA Comirnaty and inactivated CoronaVac vaccines in blood donors, Hong Kong, April 2020 to October 2021. Euro Surveill.

[83] Alhinai Z, Park S, Choe YJ, Michelow IC (2022). A global epidemiological analysis of COVID-19 vaccine types and clinical outcomes. Int J Infect Dis.

[84] Avelino-Silva VI (2016). CD4/CD8 ratio and KT ratio predict yellow fever vaccine immunogenicity in hiv-infected patients. PLoS Negl. Trop. Dis..

[85] Zhou Q (2023). Correlation between CD4 T-cell counts and seroconversion among COVID-19 vaccinated patients with HIV: A meta-analysis. Vaccines.

[86] Jin P, Li J, Pan H, Wu Y, Zhu F (2021). Immunological surrogate endpoints of COVID-2019 vaccines: The evidence we have versus the evidence we need. Signal Transduct. Target Ther..

[87] Earle KA (2021). Evidence for antibody as a protective correlate for COVID-19 vaccines. Vaccine.

[88] Garcia-Beltran WF (2021). COVID-19-neutralizing antibodies predict disease severity and survival. Cell.

[89] Coburn SB (2022). Analysis of postvaccination breakthrough COVID-19 infections among adults with HIV in the United States. JAMA Netw. Open.

