# Supplementary material for: Serological response following COVID-19 vaccines in patients living with HIV: a dose–response meta-analysis
Source: Sci Rep. 2023 Jun 19;13:9893. doi: 10.1038/s41598-023-37051-x (PMC10279716; doi:10.1038/s41598-023-37051-x)
Supplement: Supplementary file 1 — Supplementary Information. [file 41598_2023_37051_MOESM1_ESM.pdf]

Supplementary Materials

Serological response following COVID-19 vaccines in patients living with HIV: a dose-response meta-analysis

Qian Zhou, MD[1, 2], Furong Zeng, MD[3], Yu Meng, MD[1, 2], Yihuang Liu, MD[1, 2], Hong Liu, MD[1, 2*], Guangtong Deng, MD[1, 2*]

[1] Department of Dermatology, Hunan Engineering Research Center of Skin Health and Disease, Hunan Key Laboratory of Skin Cancer and Psoriasis, Xiangya Hospital, Central South University, Changsha, Hunan 410008, China

[2] National Clinical Research Center for Geriatric Disorders, Xiangya Hospital, Central South University, Changsha, Hunan 410008, China

[3] Department of Oncology, Xiangya Hospital, Central South University, Changsha, Hunan 410008, China.

[*]Correspondence to Guangtong Deng (dengguangtong@outlook.com), and Hong Liu (hongliu1014@163.com), Department of Dermatology, Hunan Engineering Research Center of Skin Health and Disease, Hunan Key Laboratory of Skin Cancer and Psoriasis, Xiangya Hospital, Central South University, Changsha, Hunan 410008, China.

This file includes:

Supplementary Fig. S1 to S28

Supplementary Tables S1 to S9

Supplementary File S1

Supplementary Figures

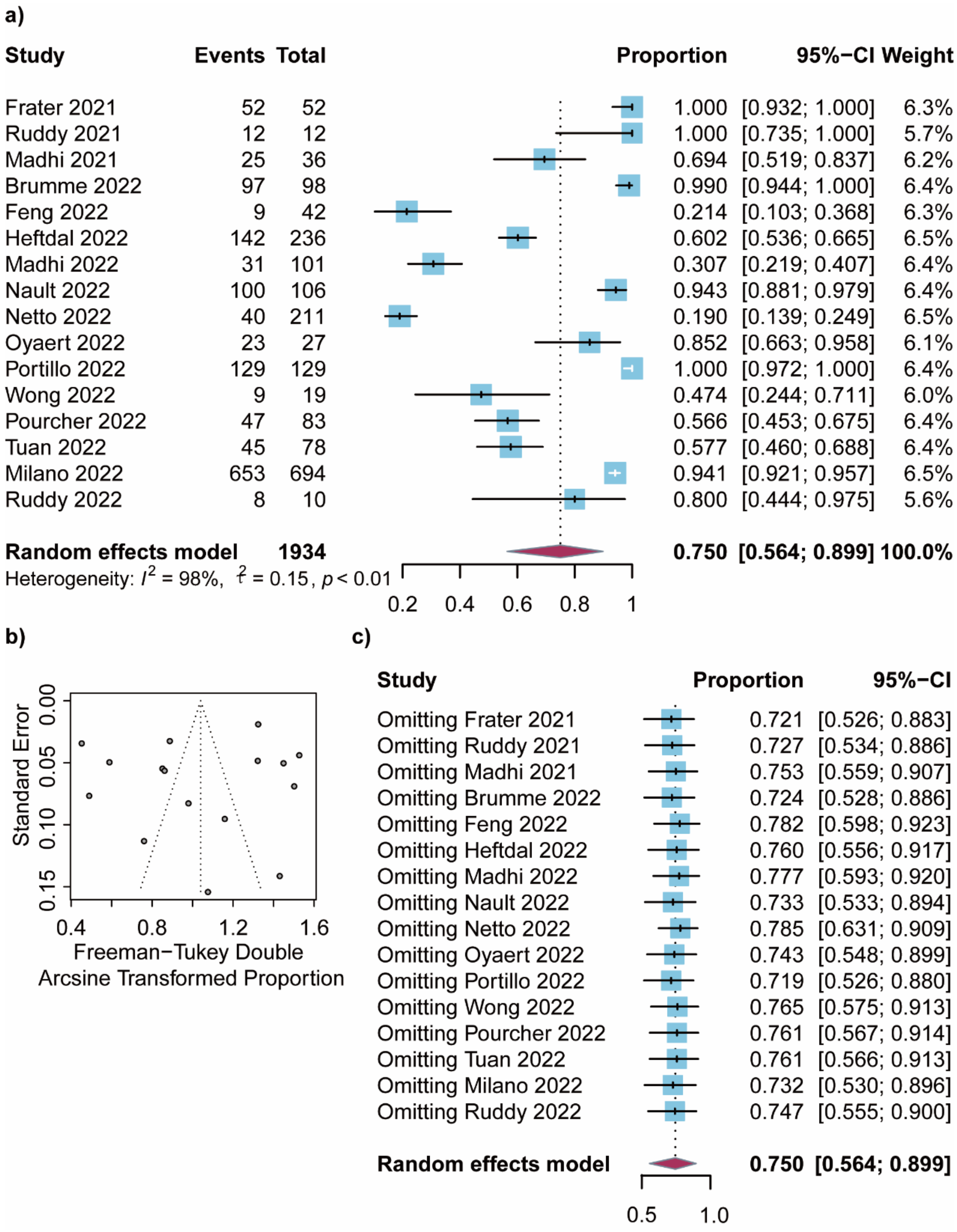

Supplementary Fig. S1. The forest plot (a), the funnel plot (b), and the sensitivity analysis using "leaving-one-out" per time approach (c) for the pooled seroconversion rate in patients with living HIV after uncomplete vaccination.

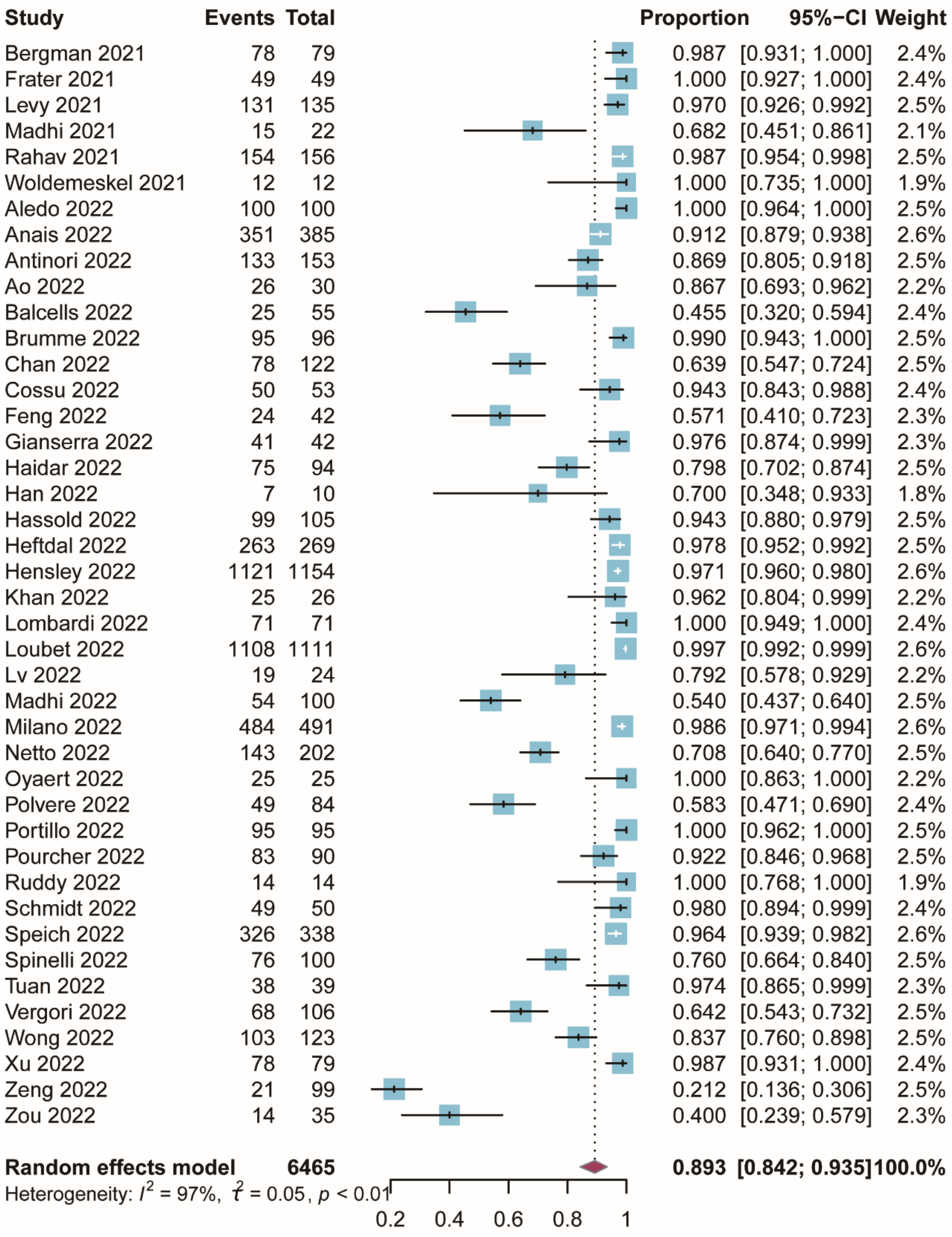

Supplementary Fig. S2. The forest plot for the pooled seroconversion rate in patients with living HIV after complete vaccination.

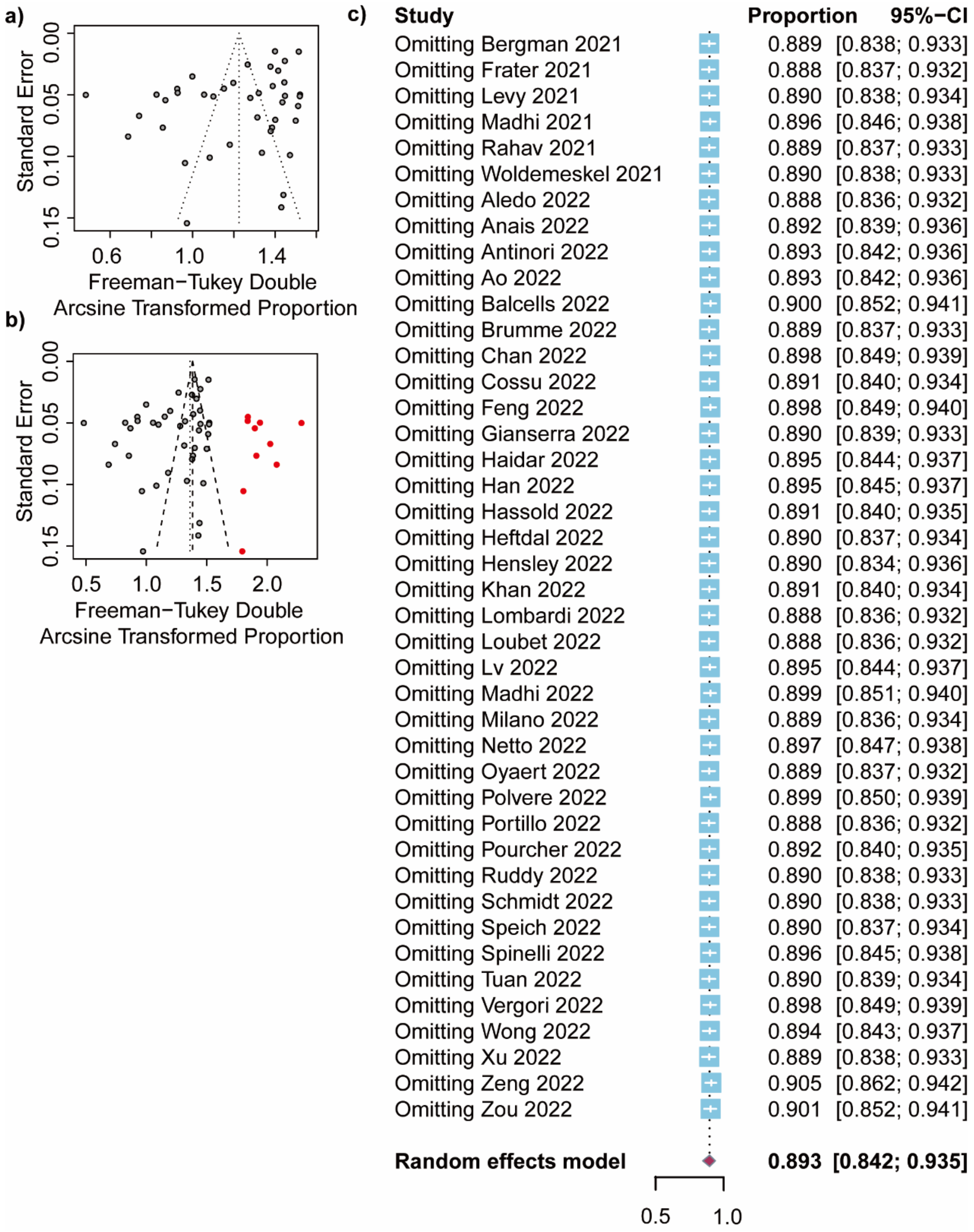

Supplementary Fig. S3. The funnel plot (a), the funnel plot after trim-and-fill analysis (b), and the sensitivity analysis using "leaving-one-out" per time approach (c) for the pooled seroconversion rate in patients with living HIV after complete vaccination.

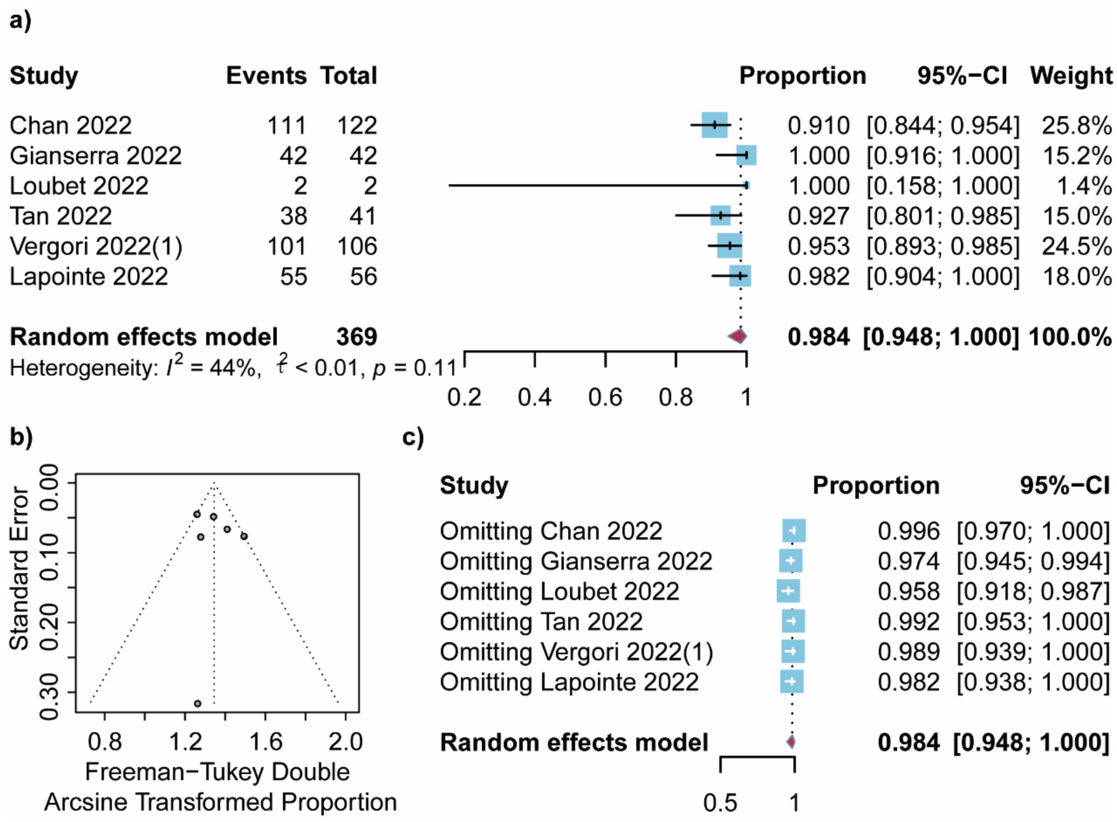

Supplementary Fig. S4. The forest plot (a), the funnel plot (b), and the sensitivity analysis using "leaving-one-out" per time approach (c) for the pooled seroconversion rate in patients with living HIV after booster vaccination.

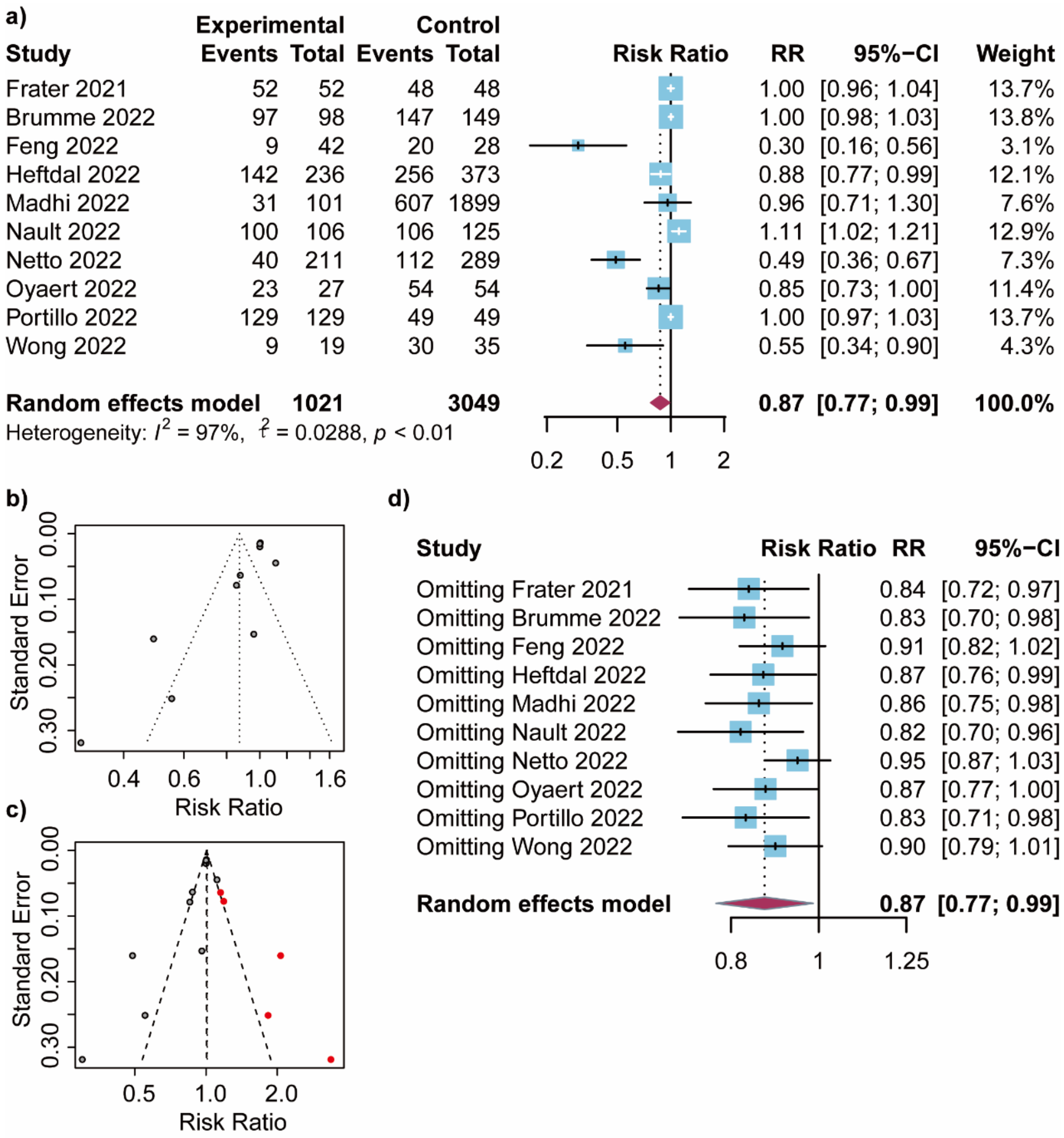

Supplementary Fig. S5. The forest plot (a), the funnel plot (b), the funnel plot after trim-and-fill analysis (c), and the sensitivity analysis using “leaving-one-out” per time approach (d) for the pooled risk ratio of seroconversion between patients with living HIV and controls after uncomplete vaccination.

Study	Experimental Events	Experimental Total	Control Events	Control Total	RR	95%−CI	Weight
Bergman 2021	78	79	78	78	0.99	[0.96; 1.01]	5.5%
Frater 2021	49	49	47	47	1.00	[0.96; 1.04]	5.2%
Levy 2021	131	135	197	201	0.99	[0.96; 1.03]	5.3%
Madhi 2021	15	22	10	15	1.02	[0.65; 1.62]	0.4%
Rahav 2021	154	156	269	272	1.00	[0.98; 1.02]	5.5%
Woldemeskel 2021	12	12	17	17	1.00	[0.87; 1.15]	2.7%
Antinori 2022	133	153	72	73	0.88	[0.82; 0.94]	4.5%
Ao 2022	26	30	26	27	0.90	[0.77; 1.05]	2.3%
Balcells 2022	25	55	54	65	0.55	[0.40; 0.75]	0.9%
Brumme 2022	95	96	150	151	1.00	[0.97; 1.02]	5.5%
Feng 2022	24	42	26	28	0.62	[0.46; 0.82]	1.0%
Haidar 2022	75	94	159	172	0.86	[0.77; 0.96]	3.3%
Han 2022	7	10	17	18	0.74	[0.49; 1.13]	0.5%
Heftdal 2022	263	269	530	538	0.99	[0.97; 1.01]	5.5%
Hensley 2022	1121	1154	422	440	1.01	[0.99; 1.04]	5.5%
Lombardi 2022	71	71	10	10	1.00	[0.87; 1.15]	2.7%
Loubet 2022	1108	1111	849	873	1.03	[1.01; 1.04]	5.6%
Khan 2022	25	26	64	73	1.10	[0.98; 1.23]	3.2%
Lv 2022	19	24	21	24	0.90	[0.70; 1.17]	1.2%
Madhi 2022	54	100	1449	1826	0.68	[0.57; 0.82]	1.9%
Netto 2022	143	202	229	274	0.85	[0.76; 0.94]	3.5%
Oyaert 2022	25	25	51	51	1.00	[0.94; 1.06]	4.6%
Polvere 2022	49	84	68	79	0.68	[0.55; 0.83]	1.7%
Portillo 2022	95	95	44	44	1.00	[0.97; 1.04]	5.3%
Schmidt 2022	49	50	56	57	1.00	[0.95; 1.05]	4.9%
Spinelli 2022	76	100	88	100	0.86	[0.76; 0.99]	2.8%
Vergori 2022	68	106	18	28	1.00	[0.73; 1.36]	0.8%
Wong 2022	103	123	25	29	0.97	[0.82; 1.15]	2.2%
Xu 2022	78	79	82	82	0.99	[0.96; 1.01]	5.5%
Zeng 2022	21	99	37	83	0.48	[0.30; 0.75]	0.4%
Zou 2022	14	35	22	38	0.69	[0.42; 1.13]	0.4%
**Random effects model**		**4686**		**5813**	**0.95**	**[0.92; 0.98]**	**100.0%**

Heterogeneity: $I^2 = 92\%$, $\tau^2 < 0.01$, $p < 0.01$

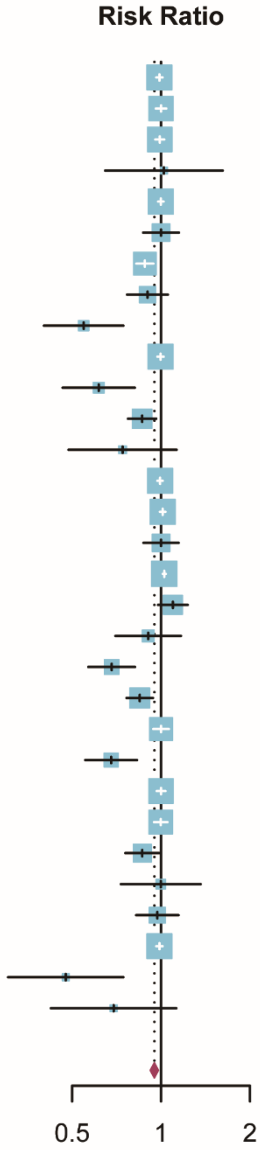

Supplementary Fig. S6. The forest plot for the pooled risk ratio of seroconversion between patients with living HIV and controls after complete vaccination.

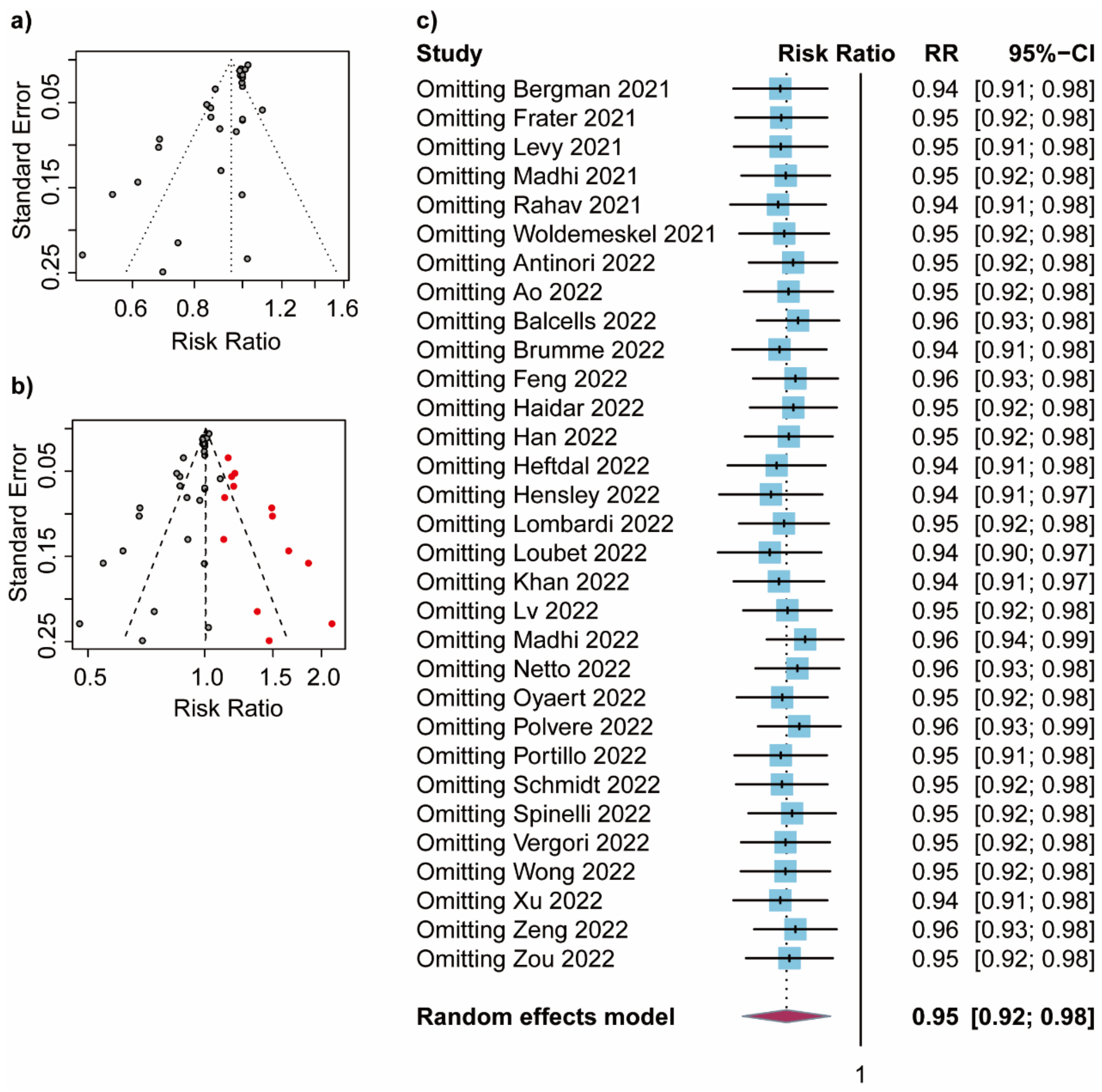

Supplementary Fig. S7. The funnel plot (a), the funnel plot after trim-and-fill analysis (b), and the sensitivity analysis using "leaving-one-out" per time approach (c) for the pooled risk ratio of seroconversion between patients with living HIV and controls after complete vaccination.

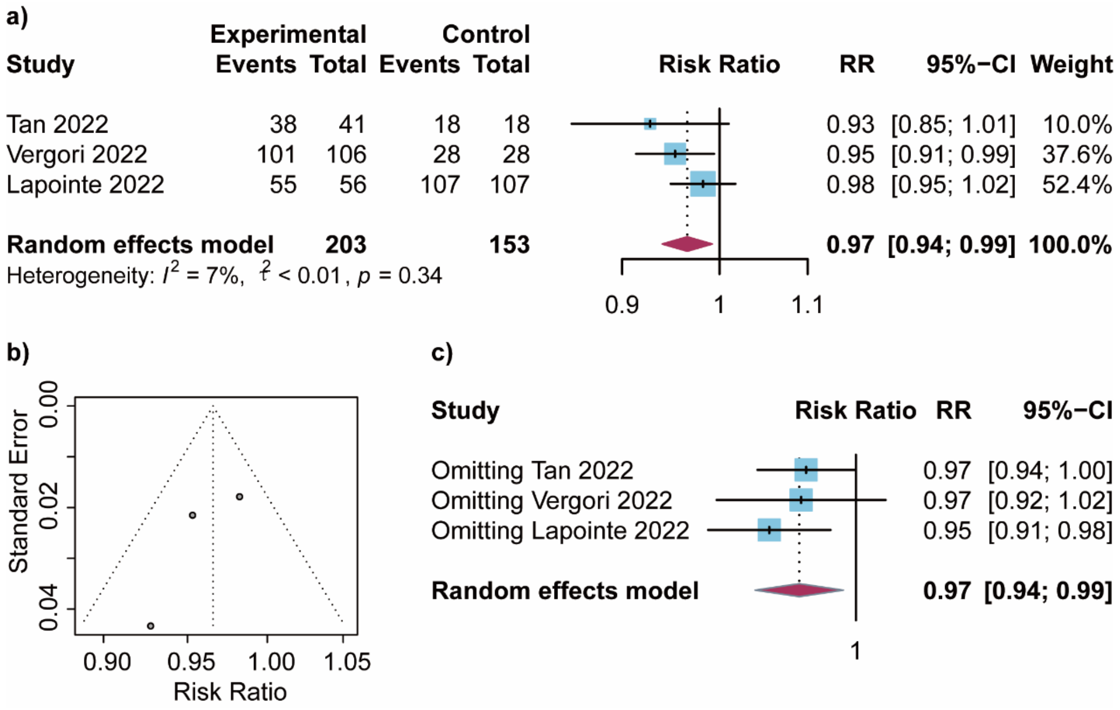

Supplementary Fig. S8. The forest plot (a), the funnel plot (b), and the sensitivity analysis using "leaving-one-out" per time approach (c) for the pooled risk ratio of seroconversion between patients with living HIV and controls after booster vaccination.

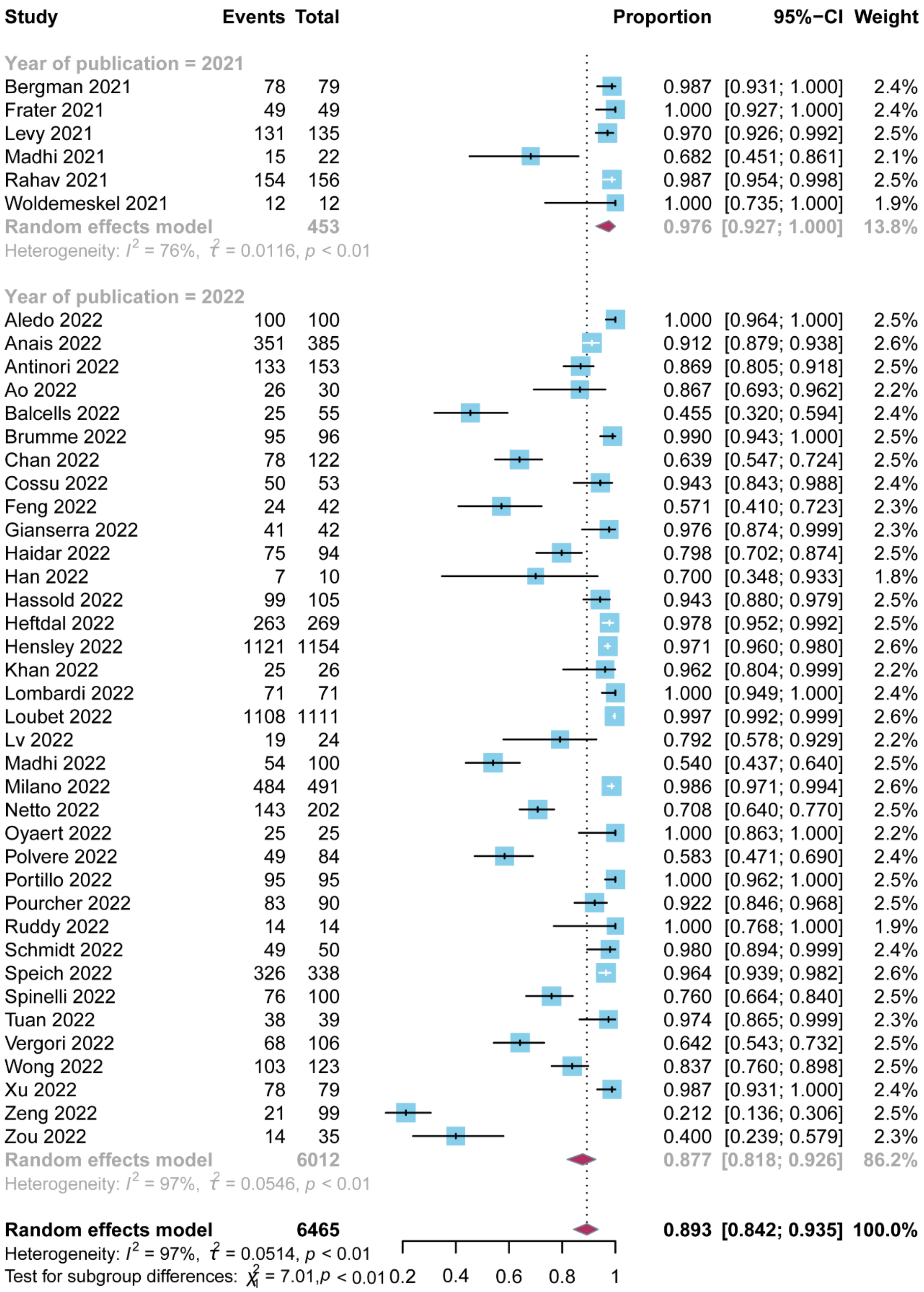

Supplementary Fig. S9. Subgroup analysis according to year of publication for the pooled seroconversion rate in patients with living HIV after complete vaccination.

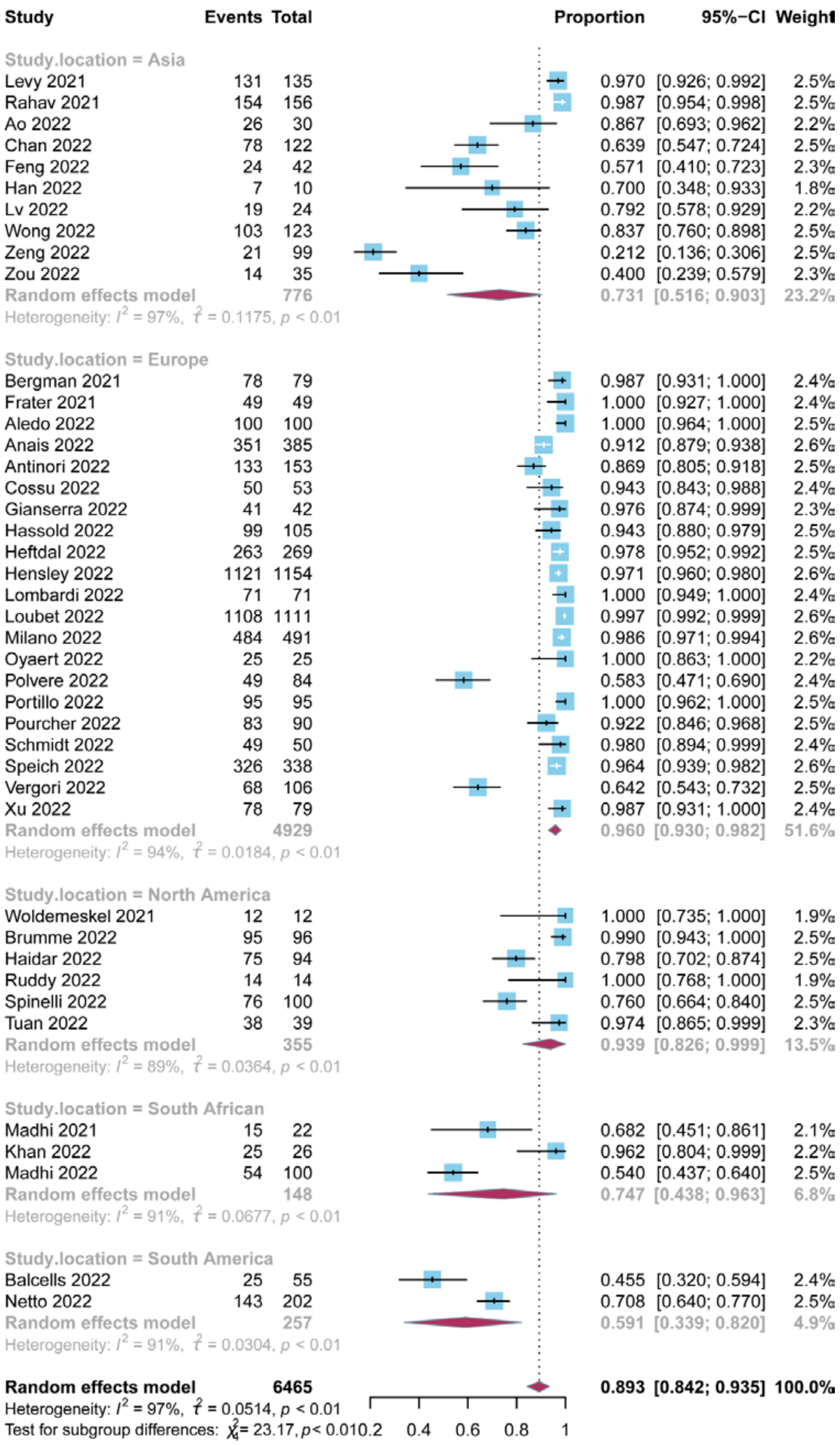

Supplementary Fig. S10. Subgroup analysis according to study location for the pooled seroconversion rate in patients with living HIV after complete vaccination.

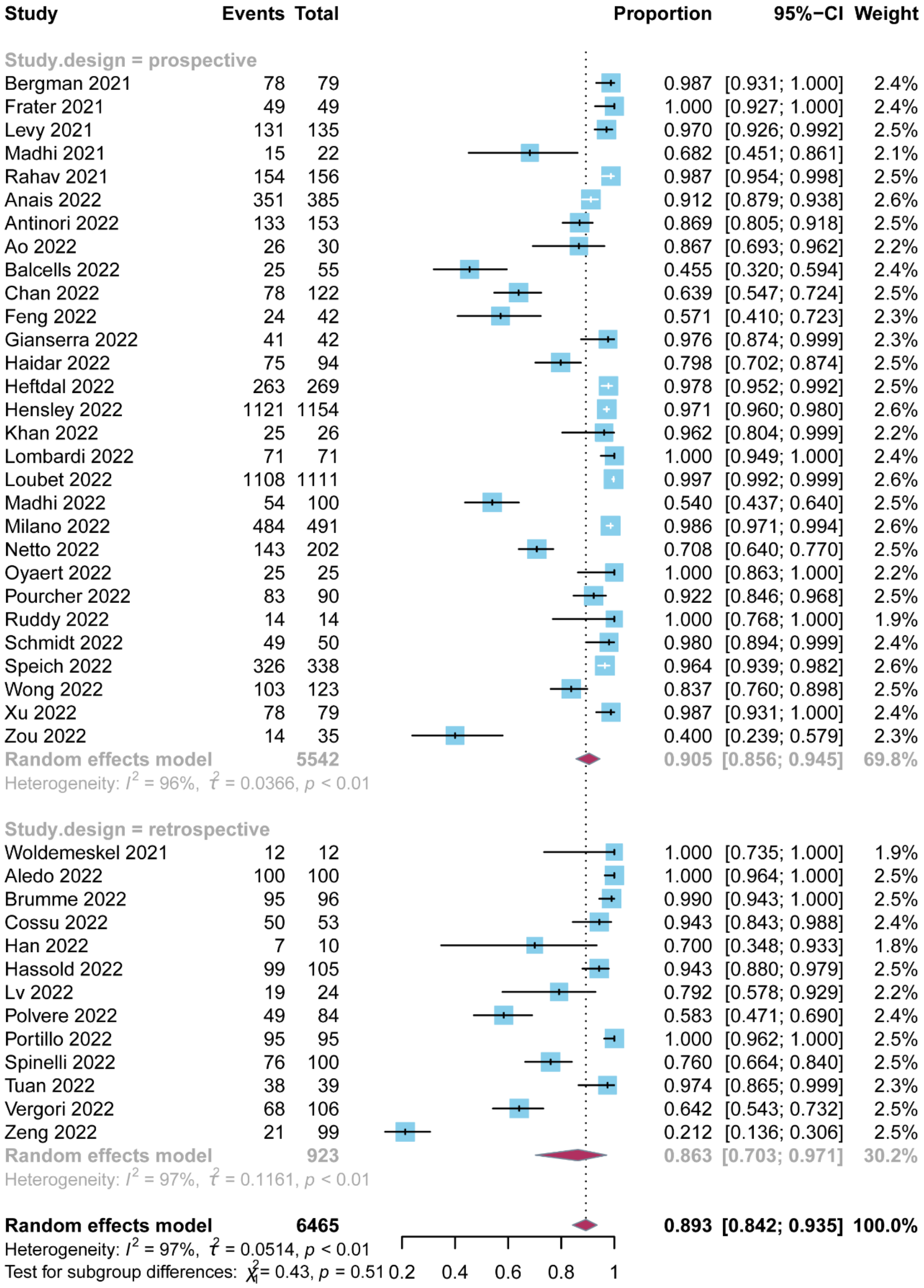

Supplementary Fig. S11. Subgroup analysis according to study design for the pooled seroconversion rate in patients with living HIV after complete vaccination.

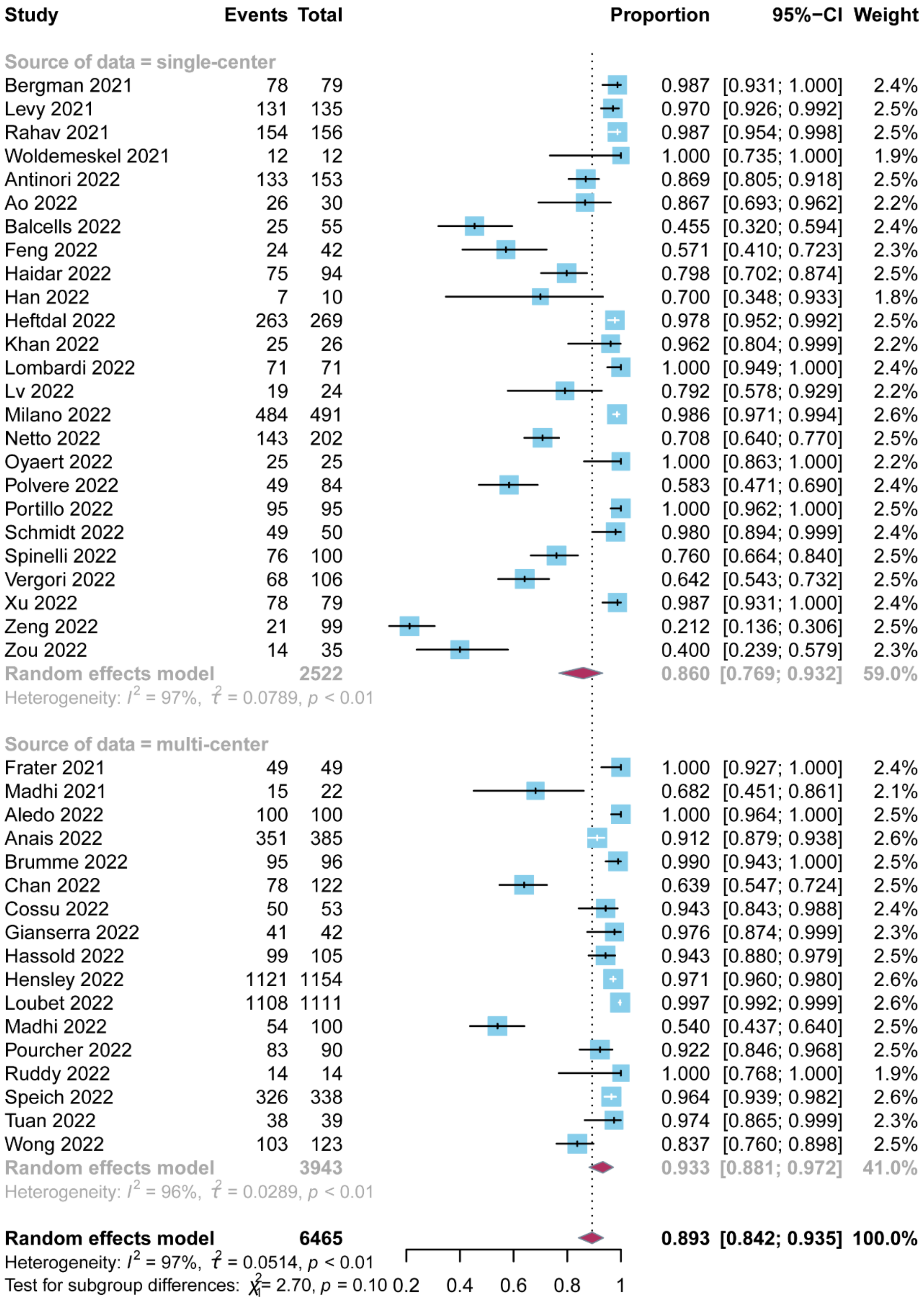

Supplementary Fig. S12. Subgroup analysis according to source of data for the pooled seroconversion rate in patients with living HIV after complete vaccination.

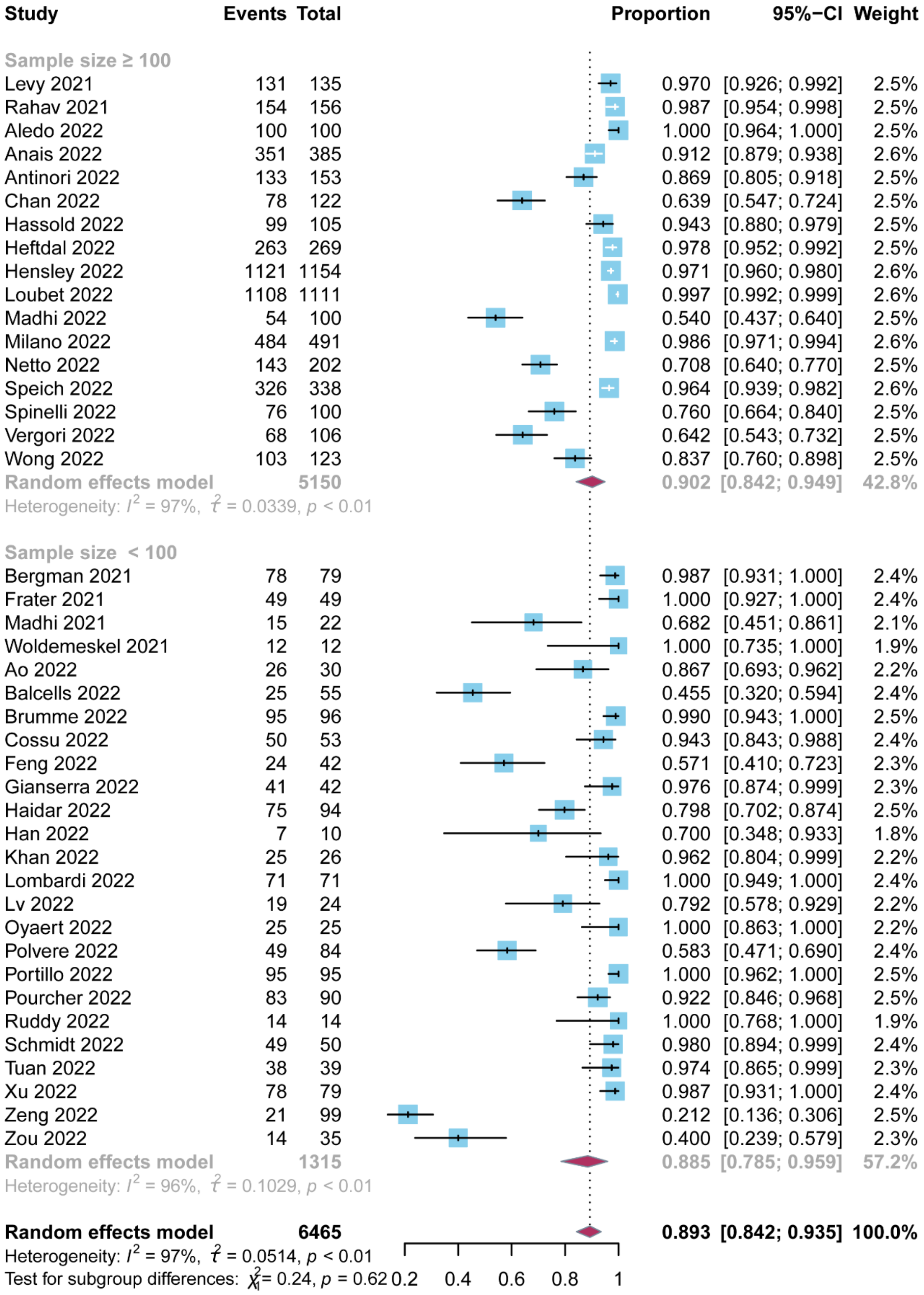

Supplementary Fig. S13. Subgroup analysis according to sample size for the pooled seroconversion rate in patients with living HIV after complete vaccination.

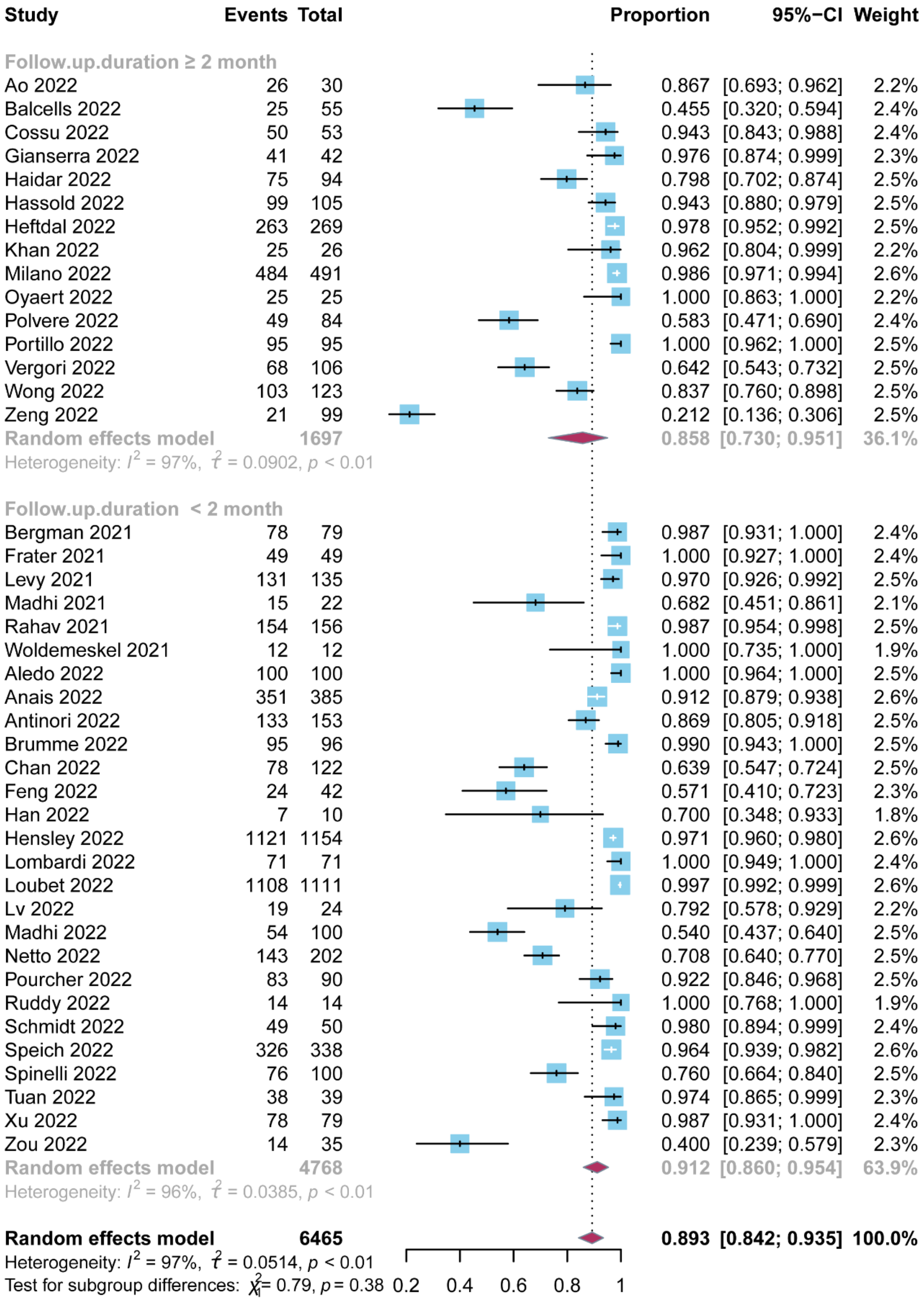

Supplementary Fig. S14. Subgroup analysis according to follow up duration for the pooled seroconversion rate in patients with living HIV after complete vaccination.

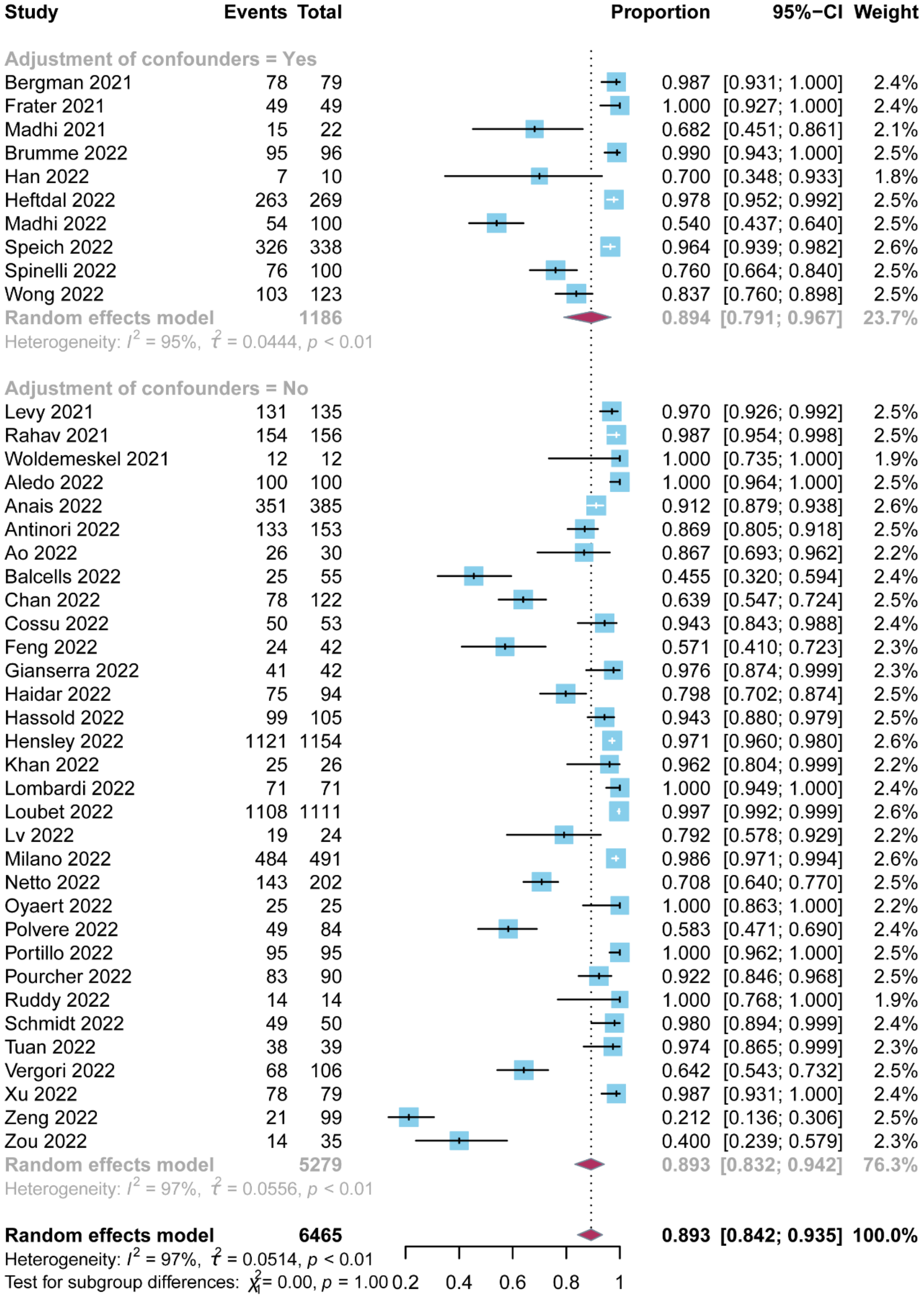

Supplementary Fig. S15. Subgroup analysis according to adjustment of confounders for the pooled seroconversion rate in patients with living HIV after complete vaccination.

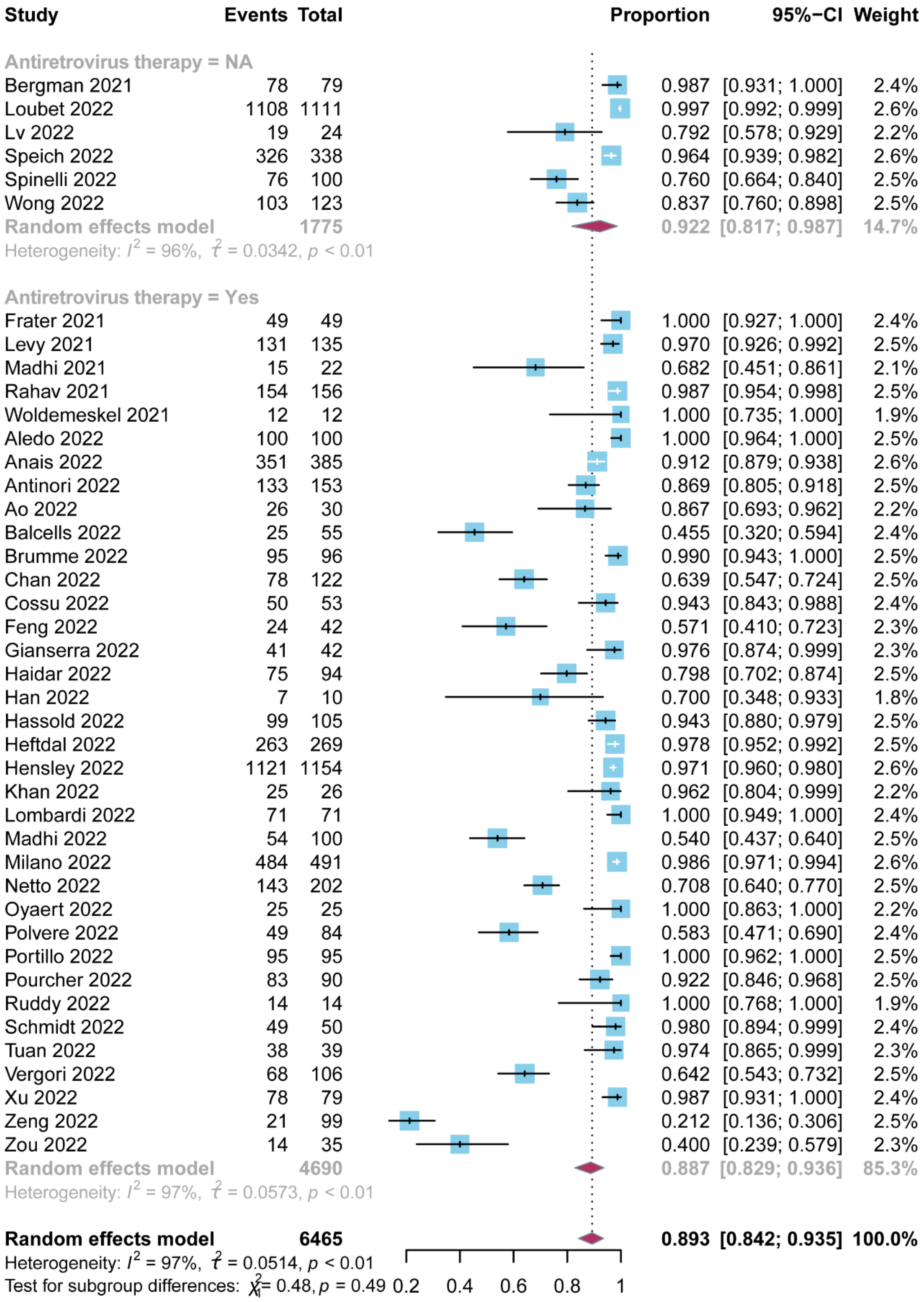

Supplementary Fig. S16. Subgroup analysis according to antiretroviral therapy for the pooled seroconversion rate in patients with living HIV after complete vaccination.

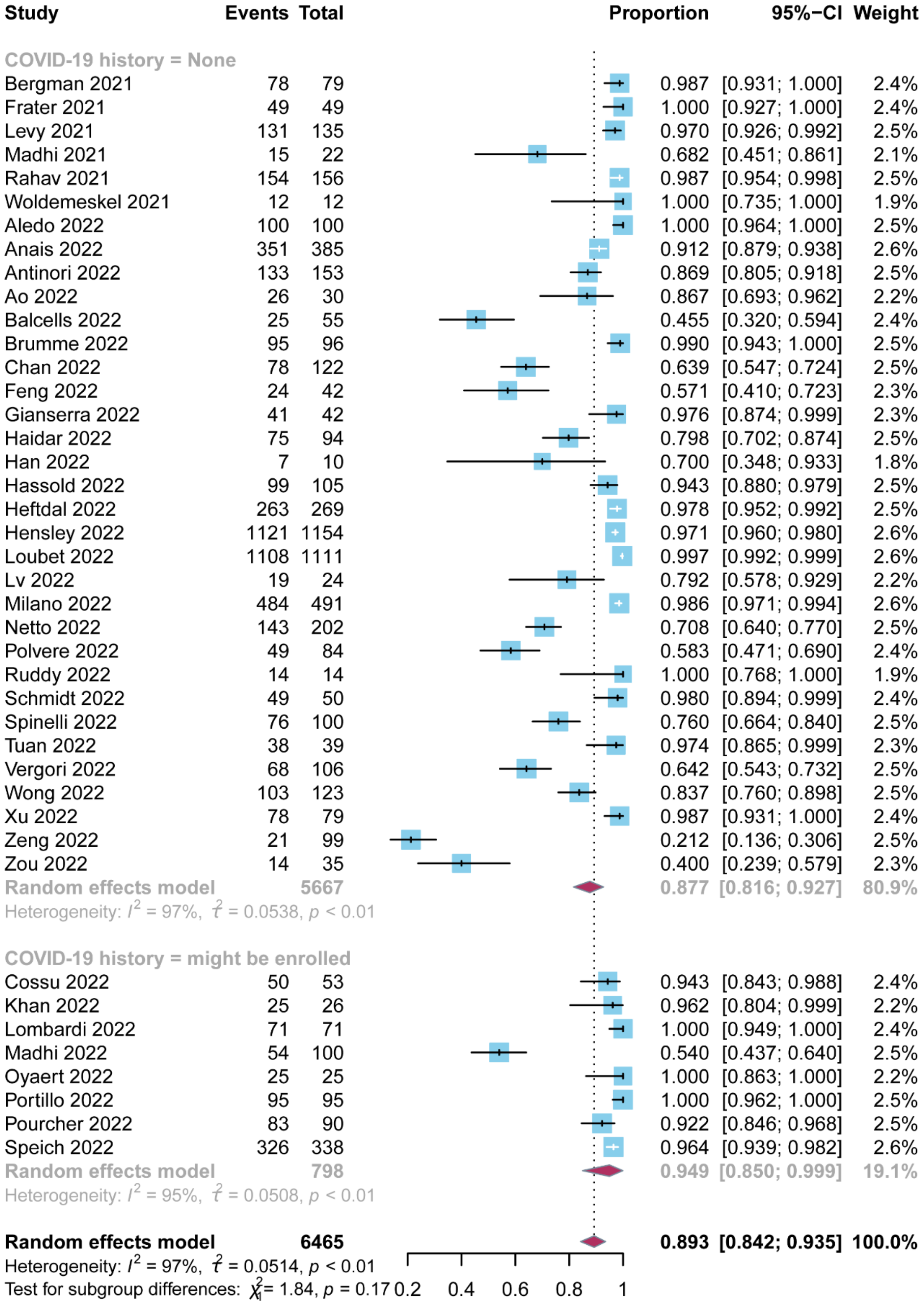

Supplementary Fig. S17. Subgroup analysis according to COVID-19 history for the pooled seroconversion rate in patients with living HIV after complete vaccination.

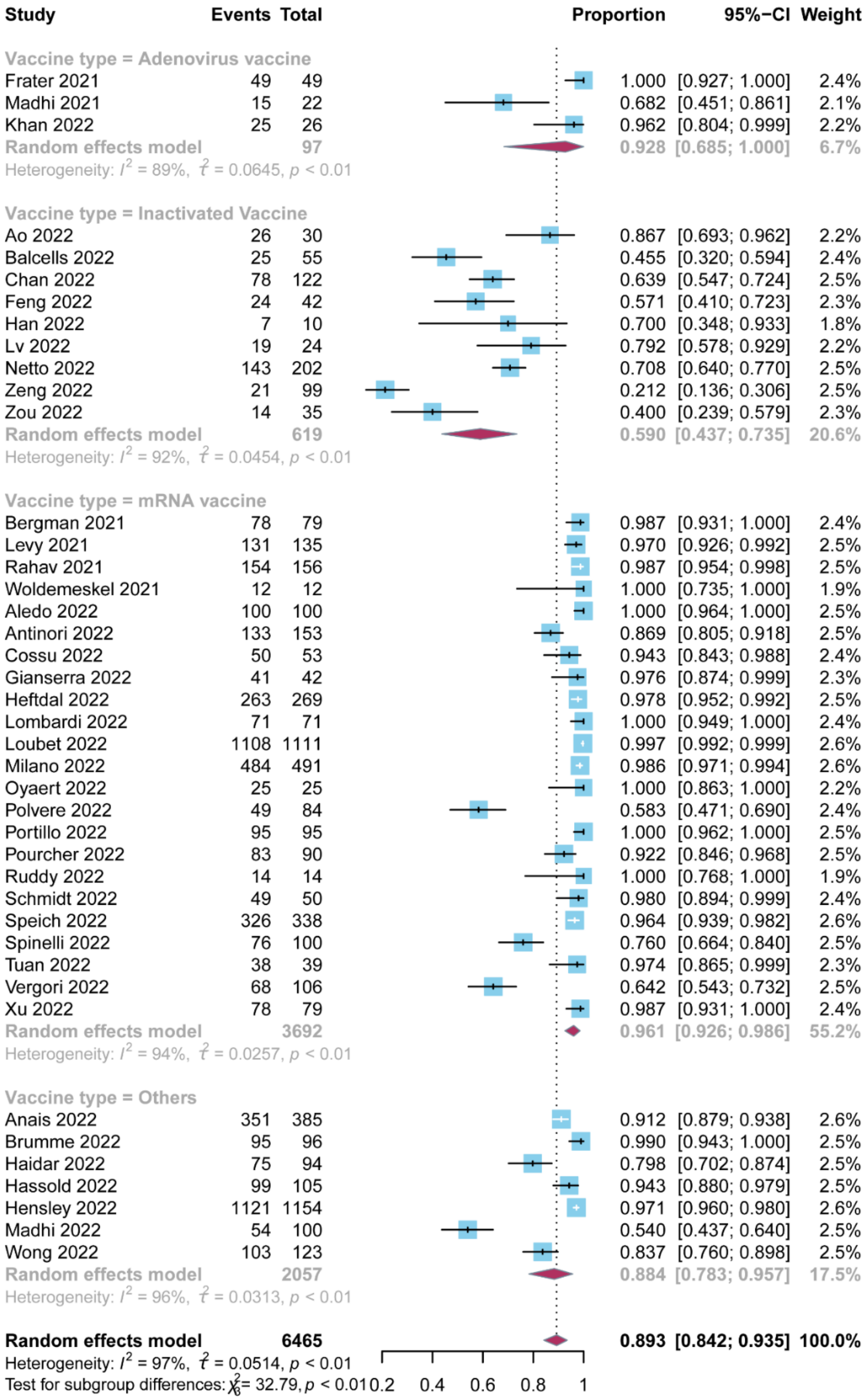

Supplementary Fig. S18. Subgroup analysis according to vaccine type for the pooled seroconversion rate in patients with living HIV after complete vaccination.

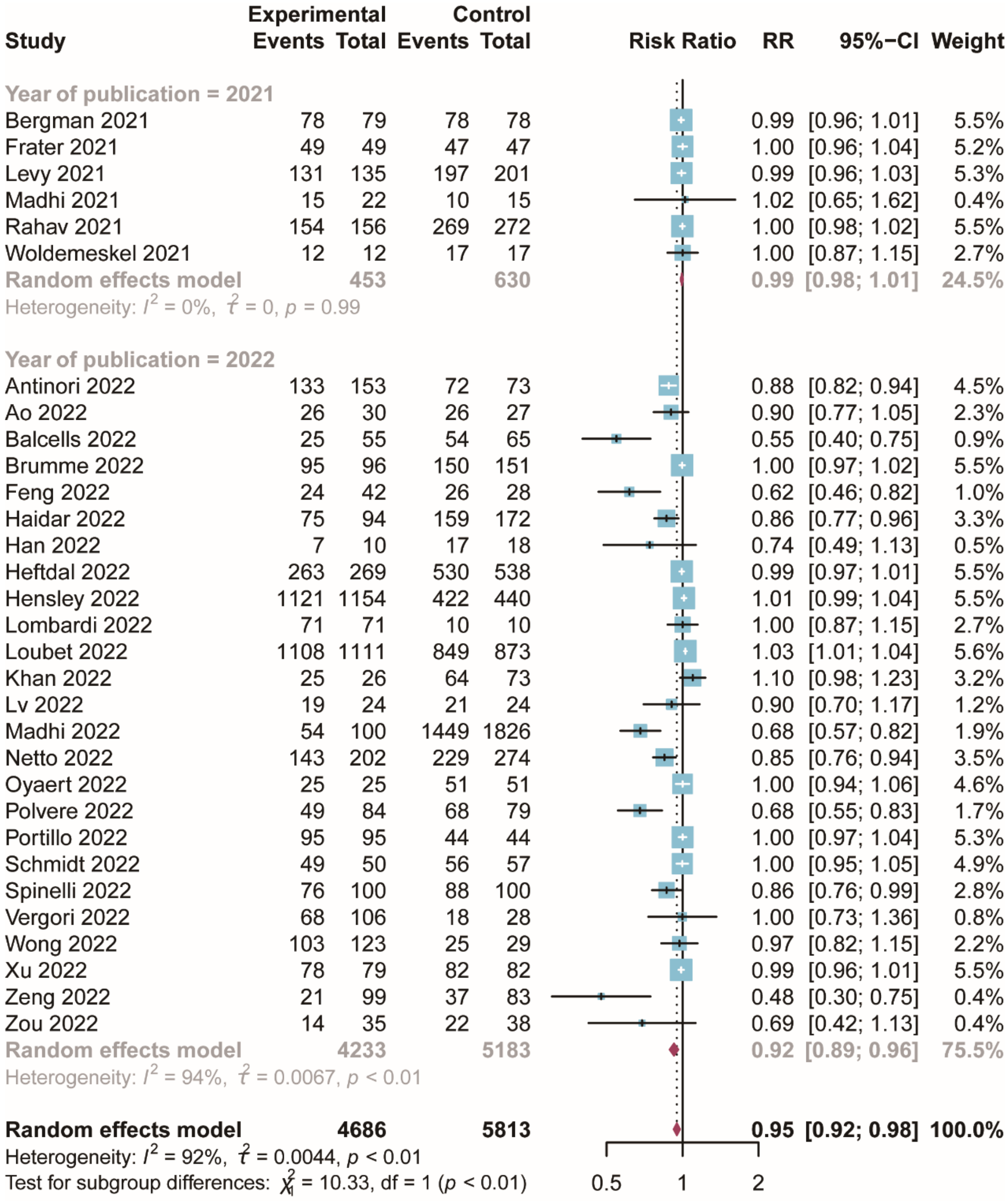

Supplementary Fig. S19. Subgroup analysis according to year of publication for the pooled risk ratio of seroconversion between patients with living HIV and controls after uncomplete vaccination.

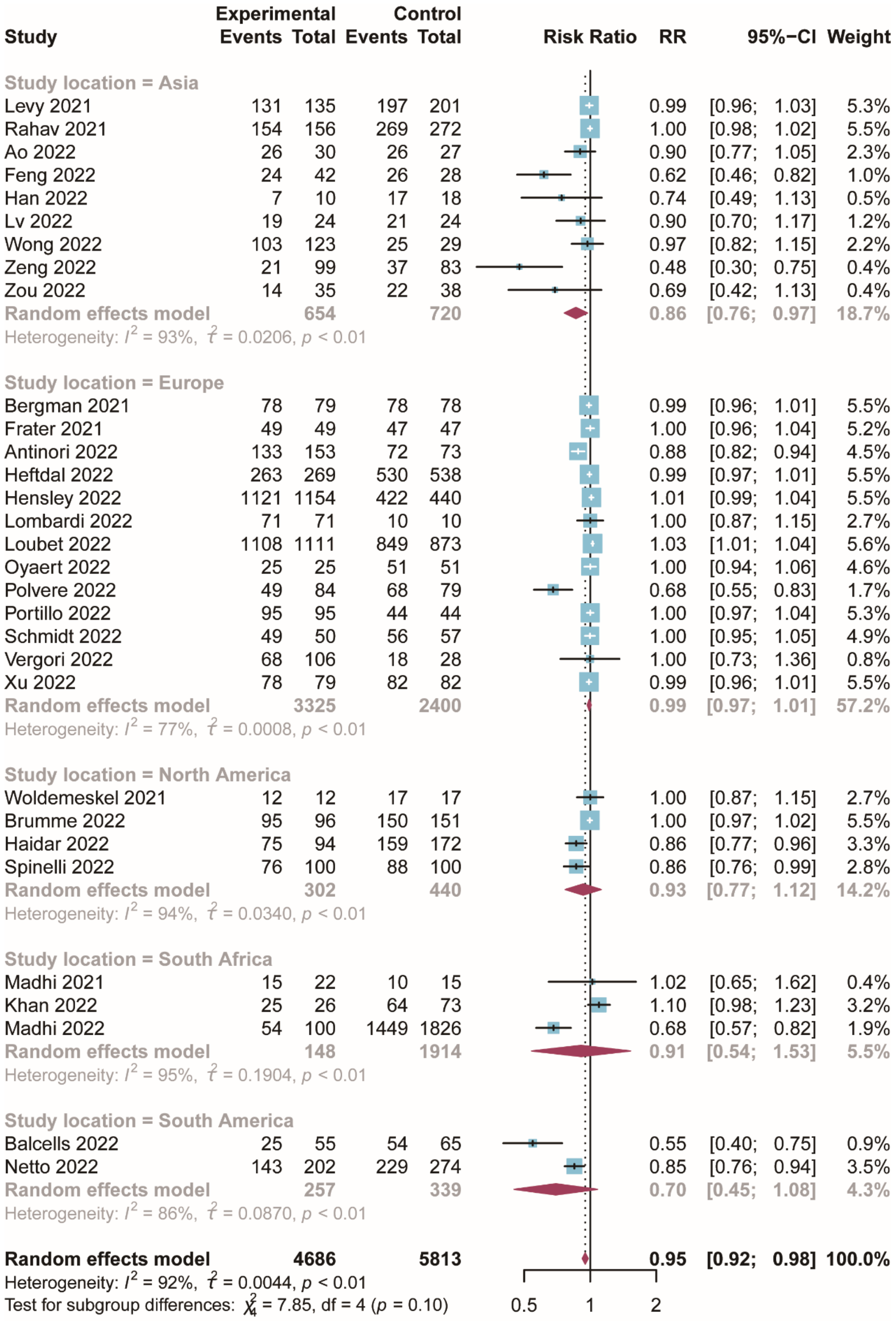

Supplementary Fig. S20. Subgroup analysis according to study location for the pooled risk ratio of seroconversion between patients with living HIV and controls after uncomplete vaccination.

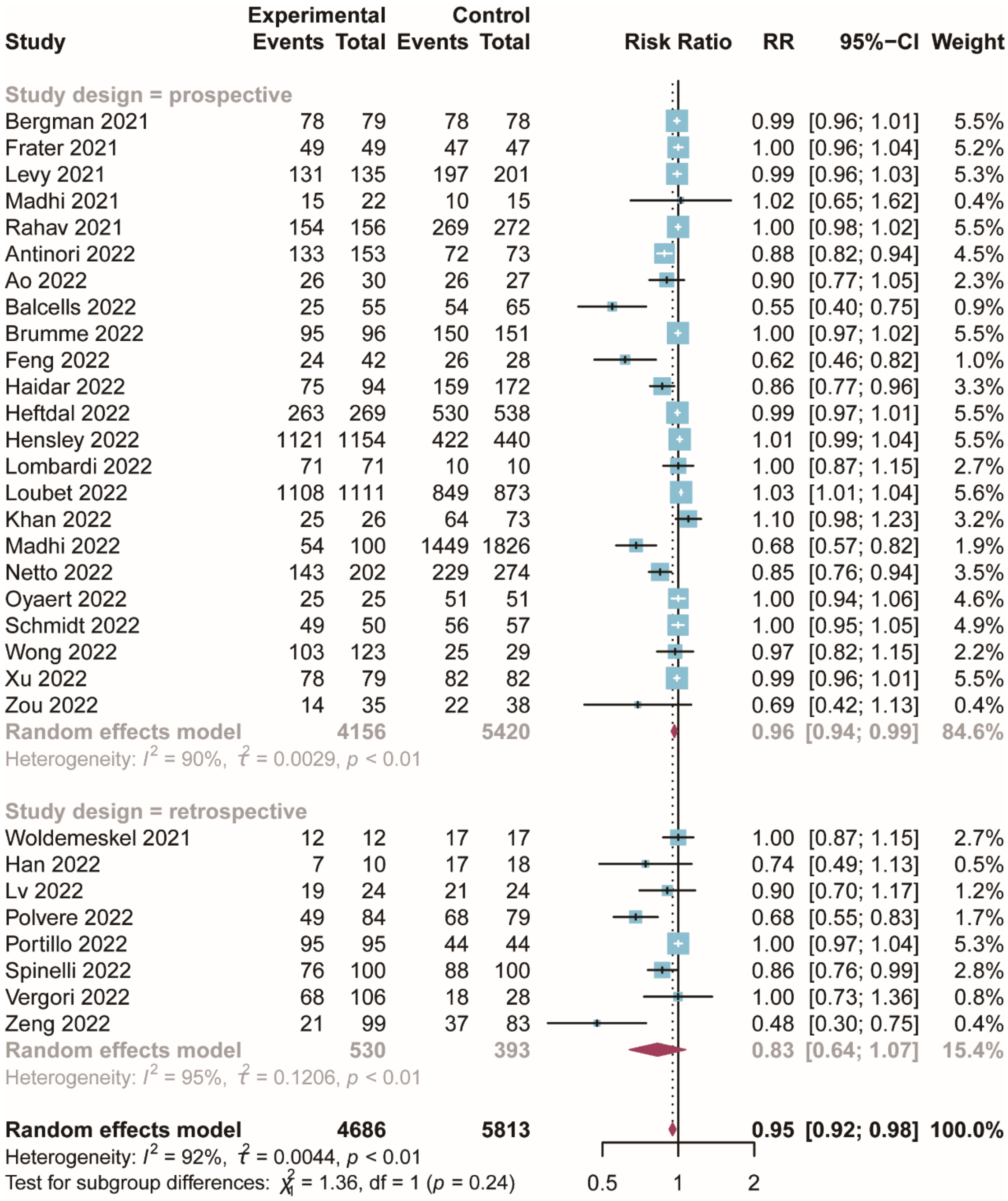

Supplementary Fig. S21. Subgroup analysis according to study design for the pooled risk ratio of seroconversion between patients with living HIV and controls after uncomplete vaccination.

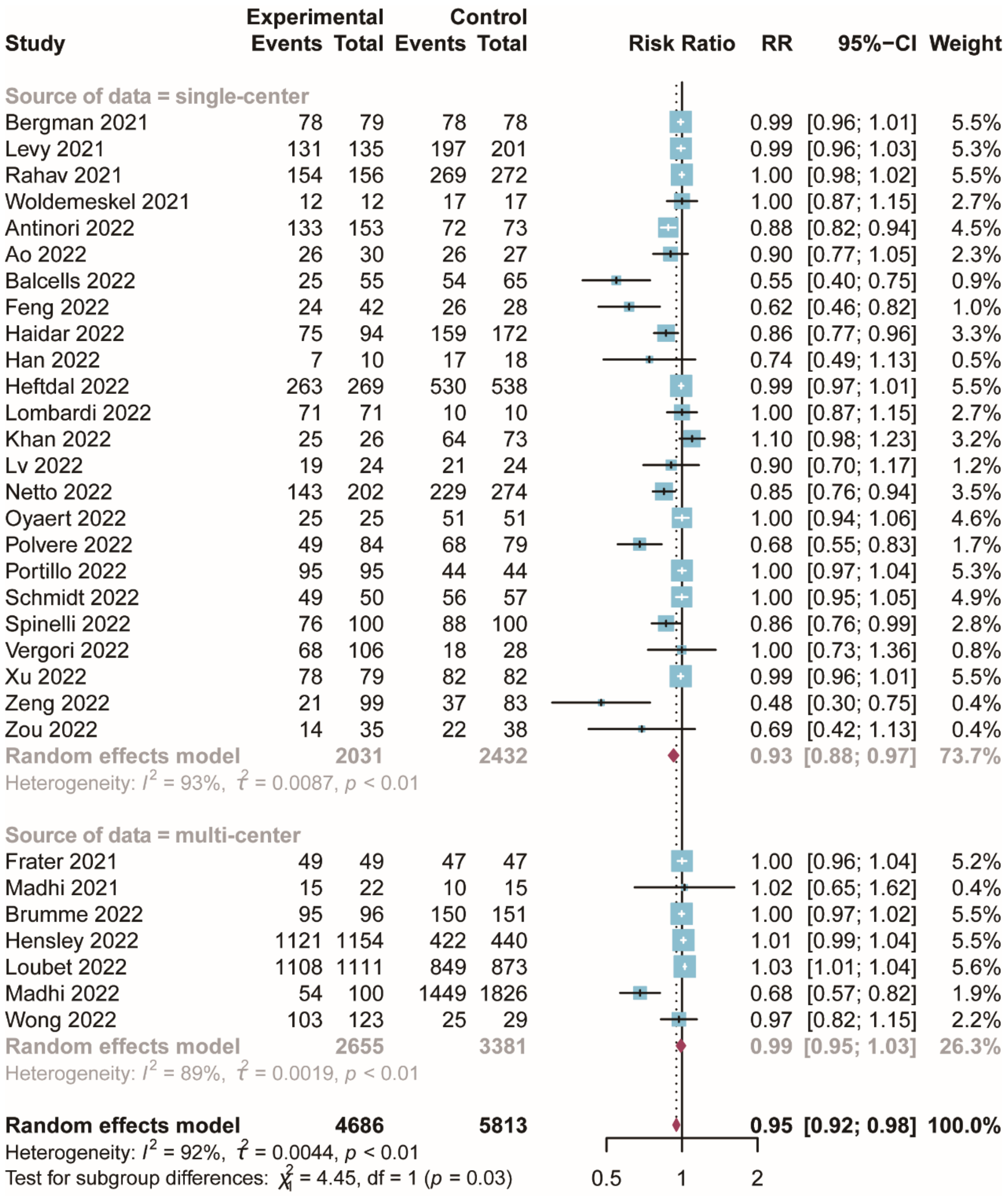

Supplementary Fig. S22. Subgroup analysis according to source of data for the pooled risk ratio of seroconversion between patients with living HIV and controls after uncomplete vaccination.

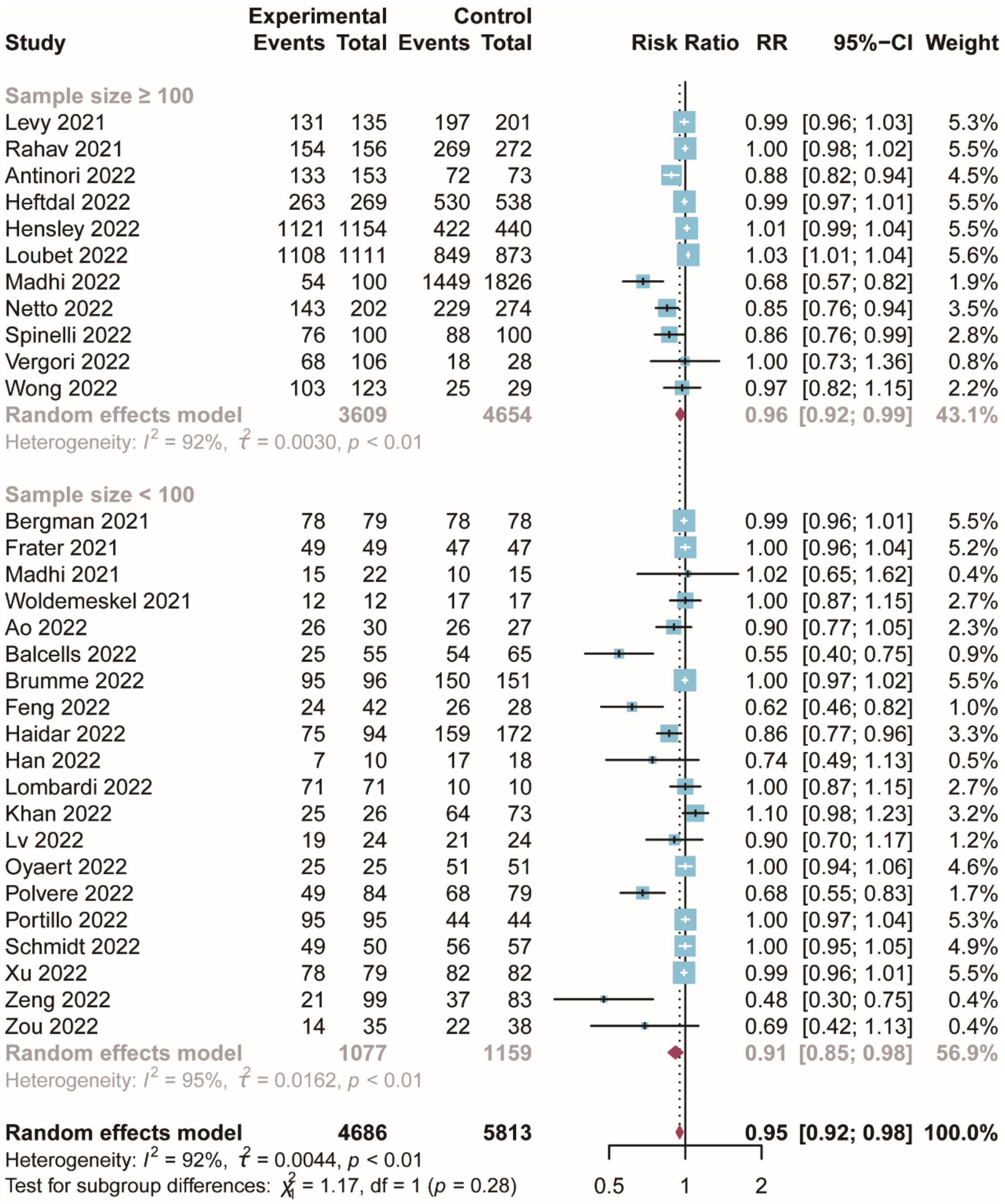

Supplementary Fig. S23. Subgroup analysis according to sample size for the pooled risk ratio of seroconversion between patients with living HIV and controls after uncomplete vaccination.

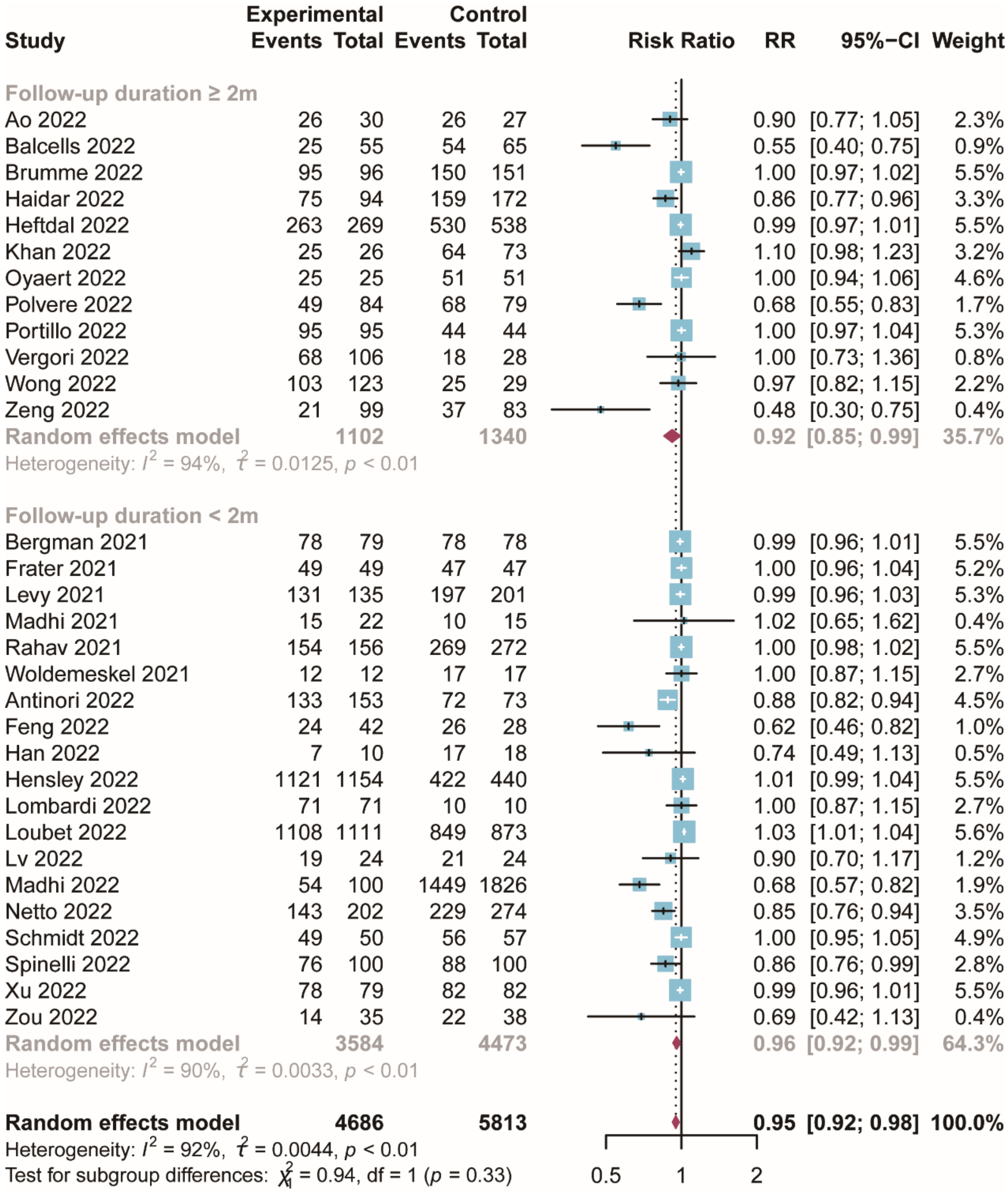

Supplementary Fig. S24. Subgroup analysis according to follow-up duration for the pooled risk ratio of seroconversion between patients with living HIV and controls after uncomplete vaccination.

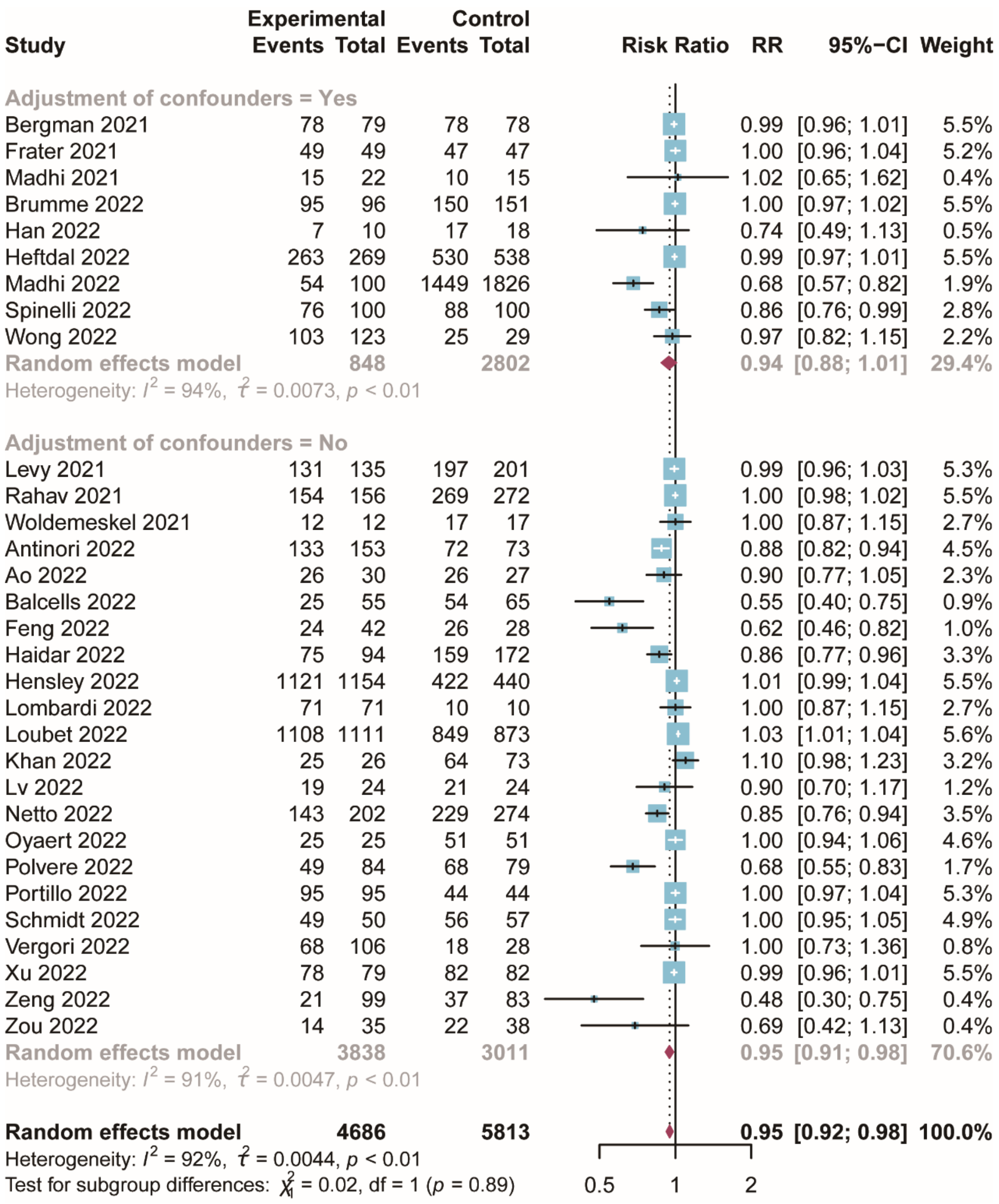

Supplementary Fig. S25. Subgroup analysis according to adjustment of confounders for the pooled risk ratio of seroconversion between patients with living HIV and controls after uncomplete vaccination.

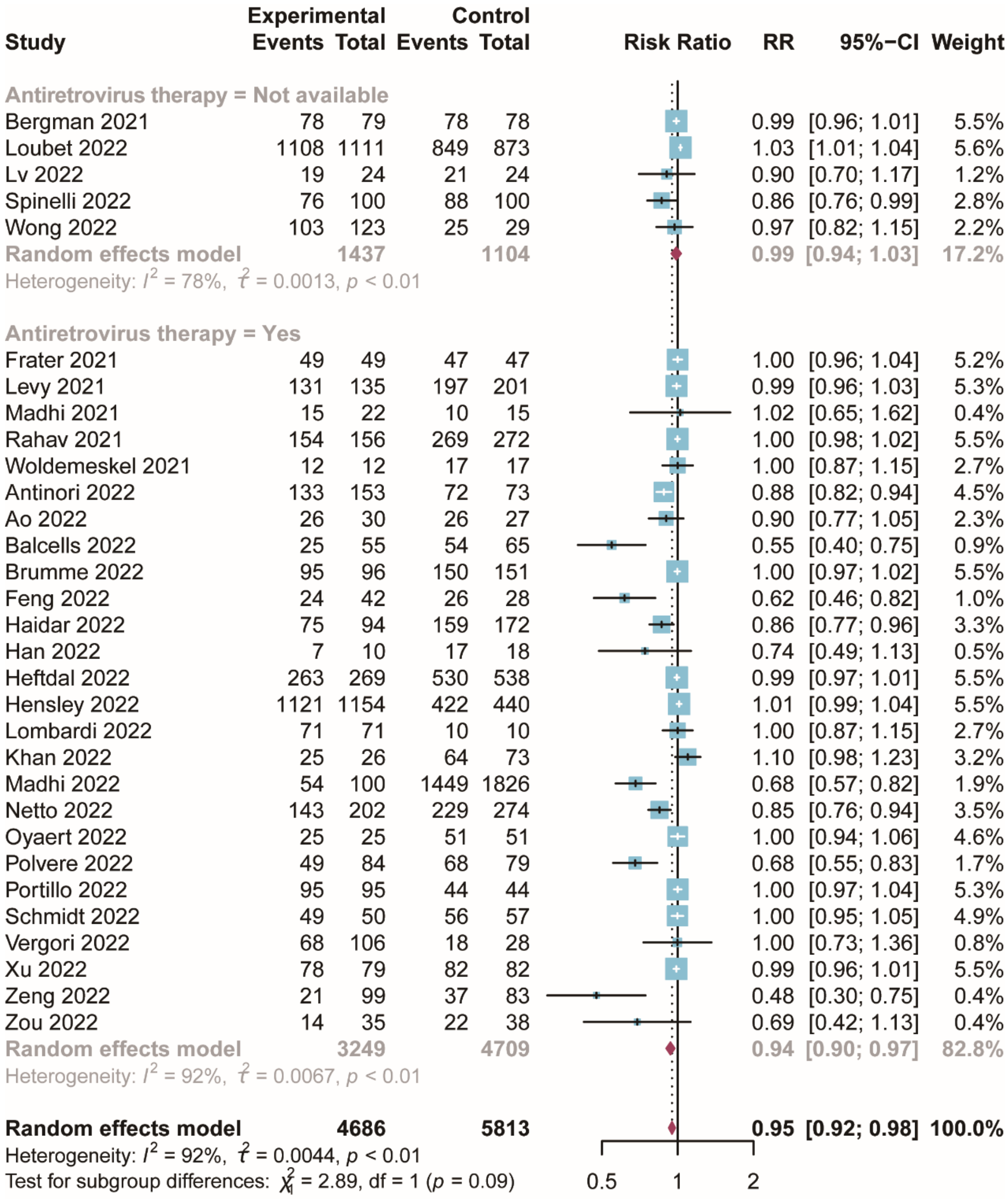

Supplementary Fig. S26. Subgroup analysis according to antiretroviral therapy for the pooled risk ratio of seroconversion between patients with living HIV and controls after uncomplete vaccination.

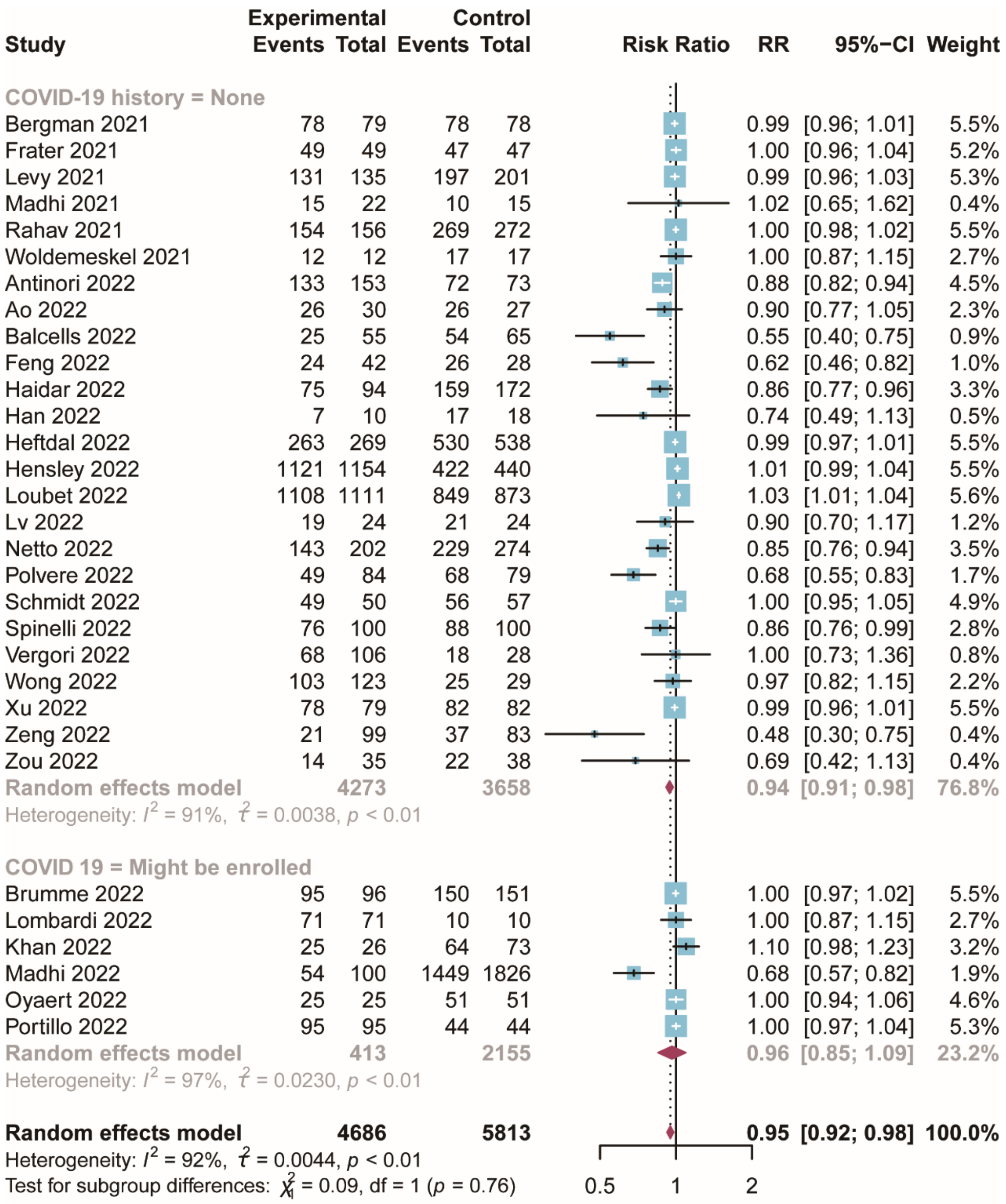

Supplementary Fig. S27. Subgroup analysis according to COVID-19 history for the pooled risk ratio of seroconversion between patients with living HIV and controls after uncomplete vaccination.

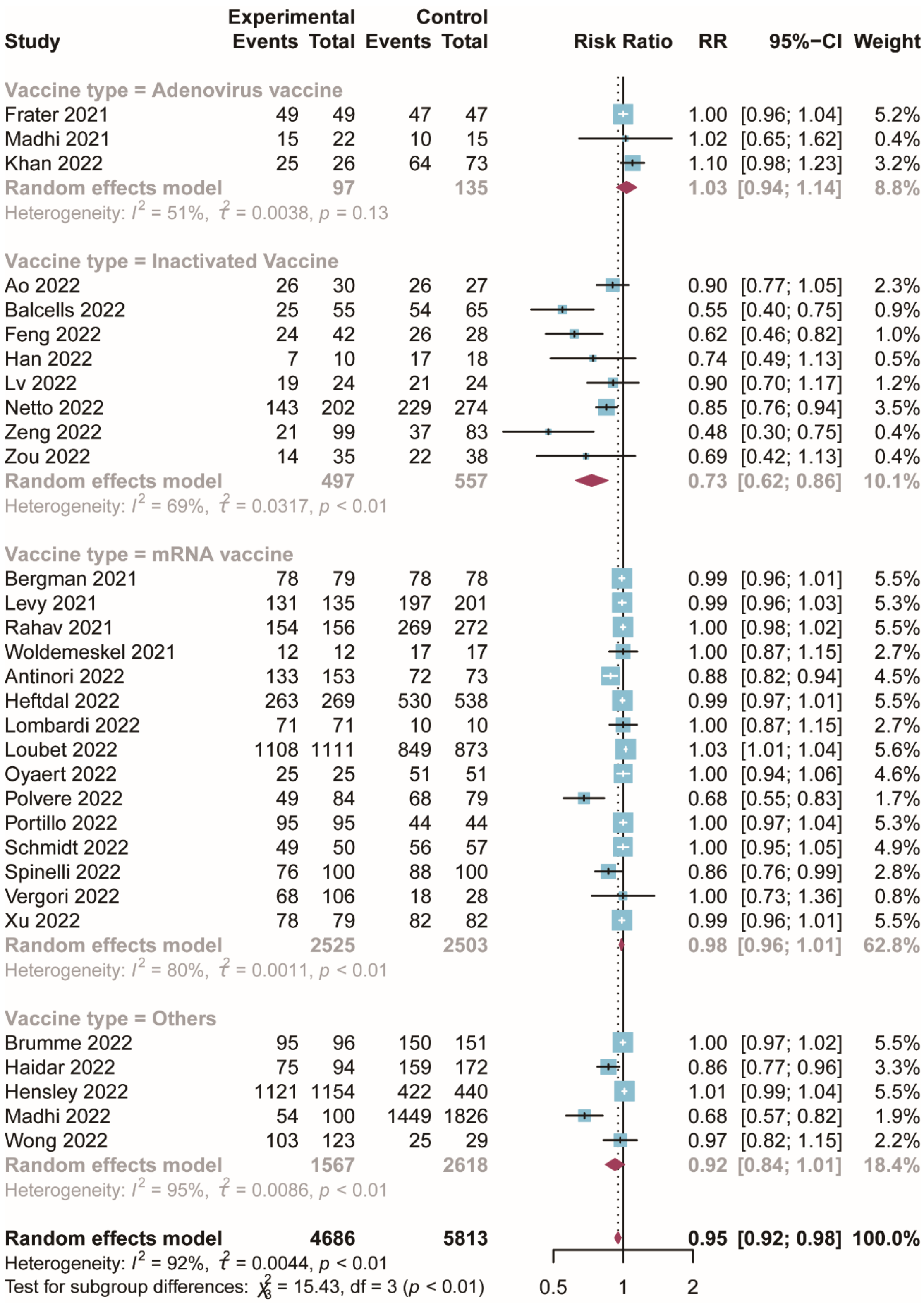

Supplementary Fig. S28. Subgroup analysis according to vaccine type for the pooled risk ratio of seroconversion between patients with living HIV and controls after uncomplete vaccination.

Supplementary Tables

Supplementary Table S1. The detailed search strategy.

Databases	Search strategies	Articles' number
PubMed	#1 corona[ti] OR covid*[ti] OR sars[ti] OR severe acute respiratory syndrome[ti] OR ncov*[ti] OR "severe acute respiratory syndrome coronavirus 2" [Supplementary Concept] OR "COVID-19" [Supplementary Concept] OR (wuhan[tiab] AND coronavirus[tiab]) OR (wuhan[tiab] AND pneumonia virus[tiab]) OR COVID19[tiab] OR COVID-19[tiab] OR coronavirus 2019[tiab] OR SARS-CoV-2[tiab] OR SARS2[tiab] OR SARS-2[tiab] OR "severe acute respiratory syndrome 2"[tiab] OR 2019-nCoV[tiab] OR (novel coronavirus[tiab] AND 2019[tiab]) NOT (animals[mesh] NOT humans[mesh])<br>#2 "Vaccines"[MeSH] OR "vaccination"[MeSH] OR vaccine[All Fields] OR vaccination[All Fields] OR vaccin*[All Fields]<br>#3 "HIV Infections" [MeSH] OR "HIV"[MeSH] OR "hiv"[tw] OR hiv infect*[tw] OR "human immunodeficiency virus"[tw] OR "human immunedeficiency virus"[tw] OR "human immuno-deficiency virus"[tw] OR "human immune-deficiency virus"[tw] OR ((human immun*) AND ("deficiency virus"[tw])) OR "acquired immunodeficiency syndrome"[tw] OR "acquired immunedeficiency syndrome"[tw] OR "acquired immuno-deficiency syndrome"[tw] OR "acquired immune-deficiency syndrome"[tw] OR ((acquired immun*) AND ("deficiency syndrome"[tw]))<br>#4 #1 AND #2 AND #3	708
Embase	#1 covid19 OR 'covid 19' OR 'sars cov 2' OR 'sars cov2' OR 'severe acute respiratory syndrome coronavirus 2' OR '2019 ncov' OR 2019ncov OR coronavirus<br>#2 'coronavirus infections'/exp<br>#3 'coronavirinae'/exp<br>#4 #1 OR #2 OR #3<br>#5 'vaccination'/exp OR vaccine OR vaccination OR vaccin*<br>#6 'human immunodeficiency virus infection'/exp OR 'human immunodeficiency virus'/exp OR 'hiv':ti,ab OR 'human immunodeficiency virus':ti,ab OR 'human immuno-deficiency virus':ti,ab OR 'human immunedeficiency virus':ti,ab OR 'human immune-deficiency virus':ti,ab OR 'acquired immune-deficiency syndrome':ti,ab OR 'acquired immunedeficiency syndrome':ti,ab OR 'acquired immunodeficiency syndrome':ti,ab OR 'acquired immuno-deficiency syndrome':ti,ab<br>#7 #4 AND #5 AND #6	603
Cochrane	#1 MeSH descriptor: [COVID-19] explode all trees	281

Library	#2 covid-19 or covid19 or covid-19 or sars-cov-2 or sars-cov2 or 'severe acute respiratory syndrome coronavirus 2'or 2019ncov or coronavirus<br>#3 MeSH descriptor: [Coronaviridae] explode all trees<br>#4 #1 OR #2 OR #3<br>#5 MeSH descriptor: [HIV Infections] explode all trees<br>#6 MeSH descriptor: [HIV] explode all trees<br>#7 hiv OR 'hiv infect*' OR 'human immunodeficiency virus' OR 'human immunedeficiency virus' OR 'human immuno-deficiency virus' OR 'human immune-deficiency virus' OR ((human immun*) AND ("deficiency virus")) OR 'acquired immunodeficiency syndrome' OR 'acquired immunedeficiency syndrome' OR 'acquired immuno-deficiency syndrome' OR 'acquired immune-deficiency syndrome' OR ((acquired immun*) AND ('deficiency syndrome'))<br>#8 #5 OR #6 OR #7<br>#9 #4 AND #8	
Duplications		-172
Total after duplications		1420

Supplementary Table S2. Characteristics of included studies.

Source	n. PLWH	Age†	Gender‡	n. controls	Age†	Gender‡	Immunoassay	Threshold for positive response
Bergman 2021	79	<65 years: 71/90 (79%)	54/90 (60%)	78	<65 years: 63/90 (70%)	39/90 (43%)	Elecsys Anti-SARS-CoV-2 S (Roche Diagnostics)	Positive serology: >0.79 U/mL
Frater 2021	52	42.5 (37.2-49.8)	54/54 (100%)	48	38.5 (29.2-45.0)	26/50 (52%)	Total IgG ELISA against trimeric SARS-CoV-2 spike protein	Positive serology: 10 EU
Levy 2021	135	Mean (SD): 49.8 (11.6)	131/143 (91.6%)	201	Mean (SD): 55.8 (14.3)	66/261 (25.3%)	SARS-CoV-2 pseudo-virus neutralization assay	Non-neutralizing: Sera not capable of reducing viral replication by 50% at 1:8 or below
Madhi 2021	26	35 (32-45)	12/37 (32·4%)	15	34 (23–42)	16/27 (59·3%)	Singleplex bead-based immunoassays were developed on the Luminex platform to quantitatively measure serum IgG binding to RBD	seroresponsive: >2-fold increase
Rahav 2021	156	49.0 (42.0-57.0)	137/156 (87.8%)	272	57.0 (44.0-67.0)	66/272 (24.3%)	VSV-spike SARS-CoV-2 pseudo-virus neutralisation assay (Gert Zimmer)	Positive serology: RBD: >1.1
Ruddy 2021	12	64 (57-70)	12/12 (100%)	-	-	-	Roche Elecsys anti-SARS-CoV-2S enzyme immunoassay	Positive serology: ≥0.80 U/mL
Woldemeskel 2021	12	Median (range): 52 (25-59)	5/12 (41.7%)	17	Median (range): 52 (25-59)	10/17 (58.8%)	Euroimmun Anti-SARS-CoV-2 IgG ELISA (Mountain Lakes, NewJersey, USA)	Not stated
Aledo	100	Mean (SD): 44 (10)	75/100	-	-	-	LIAISON SARS-CoV-2	Positive serology:

2022			(75%)				TrimericS IgG chemiluminescent assay (DiaSorin, Saluggia, Italy)	≥33.8 BAU/ml
Anais 2022	385	55 (49-60)	343/420 (82%)	-	-	-	LIAISON SARS-CoV-2 IgG kit (DiaSorin, Saluggia, Italy)	Positive serology: ≥33.8 BAU/ml
Antinori 2022	153	55 (46-59)	139/166 (83.7%)	73	42 (32–53)	48/169 (28.4%)	Microneutralization assay (MNA)	nAb titers at MNA ≥1:10
Ao 2022	30	55 (23-81)	89/139 (64%)	27	54 (21-83)	72/120 (60%)	Indirect ELISA (Sino Biological, Beijing, China)	Positive serology: OD value ≥2.1 times the mean absorance value of negative controls at 1:50 dilutions
Balcells 2022	55	Mean (range): 46.8 (52)	53/55 (96.4%)	65	Mean (range): 44.3 (51)	21/65 (32.3%)	SARS-CoV-2 surrogate virus neutralization test (sVNT) kit (GenScript)	Seropositivity: nAb titres of ≥30% at a 1:10 sample dilution
Brumme 2022	98	54 (40-61)	88/100 (88%)	151	47 (35-70)	50/152 (33%)	Roche Elecsys Anti-SARS-CoV-2 S assays	Not stated
Chan 2022	122	49 (40-56.5)	100/116 (86%)	-	-	-	Surrogate virus neutralization test	Positive serology: sVNT ≥30%
Cossu 2022	53	55 (52-62)	40/53 (75.5%)	34	41 (32-53)	8/34 (23.5%)	LIAISON SARS-CoV-2 Trimeric S IgG (DiaSorin, Saluggia, Italy)	Positive serology: ≥33.8 BAU/ml
Feng 2022	42	Mean (SD): 42.74 (10.17)	29/42 (69%)	28	Mean (SD): 37.79 (8.804)	16/28 (57.1%)	Surrogate neutralization test (Suzhou Xinbo Biotechnology Ltd, PerkinElmer, China)	Responding rates defined as ≥3-fold increase from the baseline
Gianserra	42	53 (48-61)	37/42	-	-	-	The LIAISON SARS-CoV-2	Reactive≥15 AU/mL

2022			(88.1%)				S1/S2 IgG chemiluminescent immunoassay (DiaSorin, Saluggia,Italy)	
Haidar 2022	94	Mean (SD): 57.4 (10.0)	84/94 (89.4%)	172	Mean (SD): 44.2 (13.3)	43/172 (25.0%)	Beckman Coulter SARS-CoV-2 platform (IgG against the spike protein RBD)	Reactive: ≥1.00 S/CO
Han 2022	10	34 (26-42)	45/47 (95.70%)	18	37 (33-50)	18/18 (100.0%)	SARS-CoV-2 pseudotyped virus kit (Zhongyanguobang Biological Technology Co. Ltd.)	10 or below was regarded as nonneutralizing
Hassold 2022	105	54 (46-60)	68/105 (64.8%)	-	-	-	Architect SARS-COV-2 IgG Quant II kit (Abbott,North Chicago, Illinois, USA)	Positive serology: 7.1 BAU/ml
Heftdal 2022	269	56.0 (49-64)	242/269 (90.0%)	538	56 (49-63)	73/538 (13.6%)	In-house ELISA	humoral response defined as neutralizing index ≥25%
Hensley 2022	1154	53 (44-60)	987/1154 (85.5%)	440	43 (33-53)	126/440 (28.6%)	IgG Trimeric chemiluminescence immunoassay (LIAISON, DiaSorin)	Positive serology: 33.8 BAU/ml
Khan 2022	26	39 (36-42) (Vaccinated Only); 47 (42-51) (Infected and Vaccinated)	1/26 (3.8%)	73	48 (42-55) (Vaccinated Only); 46 (40-52) (Infected and Vaccinated)	2/73 (2.7%)	Microneutralization Using the Focus-Forming Assay	Nonresponse: no detectable neutralization (FRNT50 = 1)
Lapointe 2022	56	54 (40-61)	87/99 (88%)	107	47 (35-70)	50/152 (33%)	Roche Elecsys Anti-SARS-CoV-2 S assays	Non-neutralizing: Sera not capable of reducing viral replication by 50% at 1:8 or below

Lombardi 2022	71	Mean (SD): 47 (8)	60/71 (84.5%)	10	Mean (SD): 58 (8)	7/10 (70%)	Elecsys anti-SARS-CoV-2 S (Roche Diagnostics, Monza Italy)	Not stated
Loubet 2022	1111	55.3 (49.7-61.0)	668/897 (74.5%)	873	43.9 (34.1-52.1) (< 65 years group); 70.8 (68.1-74.7) (≥ 65 years group)	543/1112 (48.8%)	Anti-SARS-CoV-2-specific neutralization assay	Seroneutralisation antibodies ≥ 20 titer
Lv 2022	24	44 (39.00-48.75)	12/24 (50.0%)	24	37 (26.25-47.25)	15/24 (62.5%)	A competitive ELISA kit to measure anti-SARS-CoV-2 neutralization antibodies	Positive: inhibition≥ 20%
Madhi 2022	101	Mean (SD): 39.0 (9.9)	37/122 (30.3%)	1899	Mean (SD): 31.5 (12.9)	1217/2089 (58.3%)	Anti-S-IgG antibodies; SARS-CoV-2 neutralising antibody assay	Positive serology: > 95% participants in the placebo group
Milano 2022	694	Median (range): 53 (19-79)	521/697 (74.7%)	-	-	-	Abbott chemiluminescent microparticle immunoassay	Positive serology: ≥50 AU/ml
Nault 2022	106	Mean (range): 43 (21-65)	90/106 (84.9%)	20	Mean (range): 47 (21-59)	7/20 (35.0%)	Anti-RBD IgG responses were measured by ELISA	Not stated
Netto 2022	211	54 (45-60)	85/215 (40%)	289	48 (37-58)	187/296 (63%)	SARS-CoV-2 sVNT RBD-HRP Kit (GenScript, Piscataway, NJ, USA)	Positive serology: at least 30%
Oyaert 2022	27	Median (range): 47 (30-66)	19/27 (70.4%)	54	Median (range): 37 (17-63)	31/54 (57.4%)	Liaison SARS-CoV-2 TrimericS IgG chemiluminescent immunoassay (CLIA) on the Liaison XL (Diasorin S.P.A., Saluggia, Italy)	33.8 BAU/mL
Polvere	84	52 (46-58)	64/84	79	52 (45-60)	22/79	cPass SARS-CoV-2 NAbs	Positive serology:

2022			(76.2%)			(27.8%)	Detection Kit, GenScript	inhibition ≥30%
Portillo 2022	129	54 (47.0-60.5)	92/131 (70.2%)	49	30 (27.0-34.0)	30/49 (61.2%)	Roche Elecsys (Roche Diagnostics,Switzerland)	Positive serology: > 1.1 IU/ml
Pourcher 2022	90	48 (39-56)	85/97 (87.6%)	-	-	-	SARS-CoV-2 Surrogate Virus Neutralization Test (Genscript)	Positive serology: inhibition≥30%
Ruddy 2022	14	62 (56-70)	13/14 (93%)	-	-	-	Roche Elecsys anti-SARS-CoV-2S enzyme immunoassay	Positive serology: ≥0.80 U/mL
Schmidt 2022	50	55 (46-60)	34/50 (68%)	57	42 (30-53)	32/60 (53.3%)	ELISA (NeutraLISA, Euroimmun)	Positive serology: ≥35%
Speich 2022	338	53 (44-61)	277/352 (78.7%)	-	-	-	The Antibody CORonavirus Assay (ABCORA) 2	Positive serology: ≥0.80 U/mL
Spinelli 2022	100	59 (50-66)	87/100 (87%)	100	59 (52-66)	87/100 (87%)	Validated surrogate virus neutralization test (sVNT)	Positive serology: ≥10 titer
Tan 2022	41	38 (33-47)	36/41 (88%)	18	32 (30-45)	10/18 (55%)	In-house SARSCoV-2 nAbs assay kit (Livzon, China)	Not stated
Tuan 2022	78	<65 years: 64/78 (82%) (uncomplete); <65 years: 33/39 (85%) (complete)	51/78 (65%) (uncomplete); 28/39 (72%) (complete)	-	-	-	Healgen (Houston, TX, USA) COVID-19 anti-S-IgG/IgM Rapid Test Cassette	Not stated
Vergori 2022	106	60 (52-68) (low CD4 nadir, CD4 <200/mm^3); 56 (47-61) (low CD4 nadir, CD4	87/106 (82.1%)	28	46.5 (34-53)	16/28 (57.1%)	Microneutralization assay (MNA)	Positive serology: ≥10 titer

		201-500/mm^3); 56 (48-60) (low CD4 nadir, CD4 >500/mm^3); 45 (42-52) (high CD4 nadir, CD4 >500/mm^3)						
Wong 2022	19	45 (35-53)	535/593 (90%)	35	37 (33-45)	42/84 (50%)	SARS-CoV-2 sVNT	Positive serology: above 20%
Xu 2022	79	53.5 (42-63.25)	54/90 (60%)	82	54 (33-67.25)	39/90 (43%)	Elecsys Anti-SARS-CoV-2 S (Roche Diagnostics International Ltd, Switzerland)	Not stated
Zeng 2022	99	Mean (SD): 35.0 (9.0) (BBIBP-CorV); 34.2 (7.9) (CoronaVac)	93/99 (93.9%)	83	Mean (SD): 33.2 (9.2) (BBIBP-CorV); 35.2 (9.2) (CoronaVac)	76/83 (91.6%)	Magnetic particle chemiluminescence kits (Shengxiang Biotechnology, Changsha, China)	Positive serology: S/CO value ≥ 1.0
Zou 2022	35	36 (31-42)	40/46 (87%)	38	31 (27-39)	19/38 (50%)	In-house SARS-CoV-2 nAbs assay kit (Zhuhai Livzon Diagnostics Inc, Zhuhai, China)	Positive serology: ≥ 10 BAU/mL.

Abbreviations: SARS-CoV-2, Severe Acute Respiratory Syndrome Coronavirus 2; PLWH, people living with HIV; ELISA, enzyme-linked immunosorbent assay; SD, Standard Deviation; RBD, receptor binding domain; EU, ELISA units; Ig, immunoglobulin; BAU, binding antibody units; AU, arbitrary units; nAb, neutralizing antibodies; sVNT, surrogate virus neutralization test; S, spike; S/CO, signal/cutoff; FRNT, focus reduction neutralization titer; IQR, interquatile range.

†Reported as median (IQR) unless otherwise stated

‡Reported as percentage of males unless otherwise stated

Supplementary Table S3. Antibody titers after the uncomplete COVID-19 vaccine.

Source	Outcome	n, PLWH	Median, unless otherwise stated	IQR, unless otherwise stated	n, controls	Median, unless otherwise stated	IQR, unless otherwise stated	Fold difference (Control/PLWH)	Endpoints of data collection
Frater 2021	Anti-S IgG	52	180 EU	87 to 410 EU	48	174 EU	129 to 364 EU	0.97	28 days after 1st dose
Madhi 2021	RBD IgG	36	GMC: 80.0 BAU/mL	40.3 to 158.8 BAU/mL	23	GMC: 51.1 BAU/mL	24.3 to 107.4 BAU/mL	0.64	28 days after 1st dose
Brumme 2022	anti-RBD antibodies	100	1.51 log10 U/mL	1.20 to 1.99 log10 U/mL	150	1.94 log10 U/mL	1.51 to 2.25 log10 U/mL	1.28	1 month after 1st dose
Cossu 2022	Anti-S IgG	53	Mean: 444 BAU/mL	SD: 665 BAU/mL	34	Mean: 478 BAU/mL	SD: 353 BAU/mL	1.08	21 days after 1st dose
Heftdal 2022	RBD IgG	269	GMC: 1079.22 AU/ml	95% CI: 897.85 to 1299.85 AU/ml	538	GMC: 1326.43 AU/ml	95%CI: 1130.03 to 1556.20 AU/ml	1.23	3 Weeks after 1st dose
Jedicke 2022	Anti-S IgG	88	18.7 RU/mL	0 to 42 RU/mL	41	20 RU/mL	12 to 27 RU/mL	1.07	Mean of 18.7 days (range 0 to 42 days) after 1st dose
Lombardi 2022	NAbs	71	182	102 to 332	10	290	81 to 6265	1.59	28 days after 1st dose
Madhi 2022	SARS-CoV-2	1899	GMT: 2068·5 EU/mL	95% CI: 1298.3 to 3295.5 EU/mL	101	GMT: 3253.5 EU/mL	95%CI: 2978.7 to 3553.6 EU/mL	1.57	21 days after 1st dose

	anti-Spike IgG								
Netto 2022	SARS-CoV-2 S1/S2 IgG	215	5.2	0.0 to 11.3	296	10.4	4.7 to 30.5	2	day 28 after 1st dose
Oyaert 2022	anti-spike (S) IgG	27	136 BAU/mL	Range: 16.4 to 9930 BAU/mL	54	495 BAU/mL	105 to 18700 BAU/mL	3.64	21 to 28 days after 1st dose
Portillo 2022	anti-RBD-IgG	129	GMT: 156.1 IU/ml	95% CI: 110.8-220.0 IU/ml	49	GMT: 308.5 IU/ml	95% CI: 206.6-460.6 IU/ml	1.98	4 weeks after 1st dose

Abbreviations: SARS-CoV-2, Severe Acute Respiratory Syndrome Coronavirus 2; PLWH, people living with HIV; IQR, interquatile range; S, spike; Ig, immunoglobulin; EU, enzyme-linked immunosorbent assay units; BAU, binding antibody units; RBD, receptor binding domain; GMC: geometric mean concentrations; GMT: geometric mean titres; SD, Standard Deviation; AU, arbitrary units; 95% CI: confidence interval; RU: relative units; IU: Internationalunit.

Supplementary Table S4. Antibody titers after the complete COVID-19 vaccine.

Source	Outcome	n, PLWH	Median, unless otherwise stated	IQR, unless otherwise stated	n, controls	Median, unless otherwise stated	IQR, unless otherwise stated	Fold difference (Control/PLWH)	Endpoints of data collection
Frater 2021	anti-spike (S) IgG	49	941 EU	531 to 1445 EU	47	631 EU	338 to 1037 EU	0.67	28 days after 2nd dose
Levy 2021	nAbs	143	GMT: 449	95% CI: 366.6 to 550.0	261	GMT: 482.8	95% CI: 410.8 to 567.5	1.08	Median of 18 days after 2nd dose
Madhi 2021	RBD IgG	32	GMC: 347·8 BAU/mL	195.0 to 620.4 BAU/mL	23	GMC: 364·2 BAU/mL	238.6 to 555.8 BAU/mL	1.05	14 days after 2nd dose
Rahav 2021	nAbs	154	GMT: 467.6	95% CI: 382.5 to 571.7	269	GMT: 474	95% CI: 403.2-557.3	1.01	2 to 4 weeks after 2nd dose
Woldemeskel 2021	SARS-CoV-2 spike binding antibodies	12	8.84	-	17	9.49	-	1.07	Median of 13 days after 2nd dose
Ao 2022	anti-RBD-IgG	30	GMT: 134.2	95% CI: 114.0 to 158.0	27	GMT: 317.5	95% CI: 267.1 to 377.4	2.37	3 months after 2nd dose
Balcells 2022	Neutralizing Activity	55	28.72%	15.74% to 54.13%	65	51.21%	34.6% to 68.6%	1.78	8 and 12 weeks (±72 hours) after 2nd dose

Bessen 2022	anti-RBD-IgG	68	GMT: 838.42 BAU/ml	95% CI: 430.79 to 1246.05 BAU/ml	20	GMT: 1841.94 BAU/ml	95% CI: 850.35 to 2833.53 BAU/ml	2.20	4 to 6 weeks after 2nd dose
Brumme 2022	Neutralizing Activity	100	40	20 to 80	152	160	70 to 320	4.00	3 months after 2nd dose
Cossu 2022	anti-spike (S) IgG	53	Mean: 309 BAU/mL	SD: 216 BAU/mL	34	Mean: 361 BAU/mL	SD: 368 BAU/mL	1.17	7 months after 1st dose
Gidari 2022	NT-Abs B.1.617.2	21	40	20 to 40	91	40	20 to 40	1.00	14 to 21 days after 2nd dose
Haidar 2022	anti-RBD-IgG S/CO level	94	4.8	1.6 to 12.5	172	5.2	2.5 to 11.5	1.08	at least 14 after 2nd dose
Han 2022	Neutralizing Activity against Delta	47	GMT: 14	95% CI: 11 to 19	18	GMT: 72	-	5.14	at least 14 after 2nd dose
Heftdal 2022	RBD IgG	269	GMC: 20442.13 AU	95% CI: 18,033.74 to 23,155.79 AU	538	GMC: 35171.05 AU	95% CI: 31,571.18 to 38,948.67	1.72	1 week after the second dose and up to 12 weeks after the first dose
Hensley 2022	anti-spike (S1) IgG	984	GMC: 1418 BAU/mL	95%CI: 1322 to 1523 BAU/mL	341	GMC: 3560 BAU/mL	95%CI: 3301 to 3840 BAU/mL	2.51	4 to 6 weeks after 2nd dose
Jedicke 2022	anti-spike (S) IgG	52	35 RU/mL	1 to 180 RU/mL	41	26 RU/mL	18 to 37 RU/mL	0.74	Mean of 35 days (range 1 to128

									days) after 2nd dose
Khan 2022	neutralization Capacity	8	GMT: 73	95% CI: 7.9 to 677	24	GMT: 6	95% CI: 2.8 to15.4	0.08	2 months after the prime dose
Lapointe 2022	Neutralizing Activity	45	20	BLOQ to 40	117	BLOQ	BLOQ to 20	-	6 months after 2nd dose
Lombardi 2022	nAbs	71	1567	789 to 3531	10	2112	719 to 8889	1.35	28 days after 2nd dose
Madhi 2022	nAbs	100	GMT: 740.3	95% CI: 508.7 to 1077.3	1941	GMT: 1188.1	95% CI: 1112.6 to 1268.7	1.60	14 days after 2nd dose
Netto 2022	nAbs	215	46.2%	26.9% to 69.7%	296	60.8%	39.8% to 79.9%	1.32	6 weeks after 2nd dose
Oyaert 2022	anti-spike (S) IgG	27	788 BAU/mL	range: 75.4 to 8860 BAU/mL	54	1320 BAU/mL	range: 104 to 8330 BAU/mL	1.68	3 months after 2nd dose
Portillo 2022	anti-RBD-IgG	96	GMT: 1303.4 IU/ml	95% CI: 1075.7 to 1579.2 IU/ml	44	GMT: 1896.5 IU/ml	95% CI: 1611.4 to 2232.1 IU/ml	1.46	5 months after 2nd dose
Schmidt 2022	Neutralizing Activity	50	98.7%	92.2% to 99.4 %	57	99%	97.6 to 99.4%	1.00	7 to 155 days after 2nd dose
Tau 2022	anti-RBD-IgG	136	118 BAU/mL	61.2 to 238.6 BAU/mL	61	101.4 BAU/mL	52.5 to 185 BAU/mL	0.86	3 to 6 months months after 2nd dose
Xu 2022	SARS-CoV-2 spike IgG	79	1613 U/ml	897 to 2643 U/ml	82	2192 U/ml	1398 to 3651 U/ml	1.36	14 days after 2nd dose

Zou 2022	nAbs	46	GMC: 8.07 BAU/mL	95% CI: 5.67 to 11.48 BAU/mL	36	GMC: 11.09 BAU/mL	95% CI: 6.68 to 18.42 BAU/mL	1.37	42 days after 2nd dose

Abbreviations: SARS-CoV-2, Severe Acute Respiratory Syndrome Coronavirus 2; PLWH, people living with HIV; IQR, interquatile range; S, spike; Ig, immunoglobulin; EU, enzyme-linked immunosorbent assay units; nAb, neutralizing antibodies; BAU, binding antibody units; RBD, receptor binding domain; GMC: geometric mean concentrations; GMT: geometric mean titres; SD, Standard Deviation; S/CO, signal/cutoff; 95% CI: confidence interval; AU, arbitrary units; RU: relative units; IU: Internationalunit; BLOQ: below the limit of quantitation.

Supplementary Table S5. Antibody titers after the boost COVID-19 vaccine.

Source	Outcome	n, PLWH	Median, unless otherwise stated	IQR, unless otherwise stated	n, controls	Median, unless otherwise stated	IQR, unless otherwise stated	Fold difference (Control/PLWH)	Endpoints of data collection
Lapointe 2022	Neutralizing Activity	56	640	160 to1280	107	320	160 to 320	0.5	1 moth after 3rd dose
Tan 2022	nAbs against RBD antigen	41	GMT: 39.010 BAU/mL	95%CI: 21.380 to 71.160 BAU/mL	18	GMT: 100.800 BAU/mL	95%CI: 40.280 to 252.300 BAU/mL	2.58	14 days after 3rd dose
Vergori 2022	nAbsT against BA.1	25	Mean: 6.4 log2	SD 1.3	28	Mean: 6.1 log2	SD 1.8	0.95	2 weeks after 3rd dose

Abbreviations: PLWH, people living with HIV; IQR, interquatile range; nAb, neutralizing antibodies; BAU, binding antibody units; RBD, receptor binding domain; GMT: geometric mean titres; SD, Standard Deviation; 95% CI: confidence interval;

Supplementary Table S6. Risk of bias of all included studies.

(a) Risk of bias of randomized controlled trails (Cochrane Risk of Bias 2 Tool).

Studies (year)	Sequence generation	Allocation concealment	Blinding of participants	Blinding of outcome assessors	Incomplete data	Selective outcomes reporting	Other sources	Overall bias
Madhi 2021	L	L	L	L	L	L	L	L
Madhi 2022	L	L	L	Some concerns	L	L	L	M
Speich 2022	L	L	High	High	L	L	L	High

(b) Risk of bias of cohort studies using the ROBINS-I scale.

Studies (year)	Confounding	Participant selection	Classification of interventions	Deviations from intended interventions	Missing data	Outcome measurement	Selective outcome reporting	Overall bias
Bergman 2021	S	L	L	L	L	L	L	S
Frater 2021	S	L	L	L	L	L	L	S
Levy 2021	C	L	L	L	L	L	L	C
Rahav 2021	C	L	L	L	L	L	L	C
Woldemeskel 2021	C	L	L	L	L	L	L	C
Antinori 2022	C	L	L	L	L	L	L	C
Ao 2022	C	L	L	L	L	L	L	C
Balcells 2022	C	L	L	L	L	L	L	C
Brumme 2022	S	L	L	L	L	L	L	S

Feng 2022	C	L	L	L	L	L	L	C
Haidar 2022	C	L	L	L	L	L	L	C
Han 2022	S	L	L	L	L	L	L	S
Heftdal 2022	S	L	L	L	L	L	L	S
Hensley 2022	C	L	L	L	L	L	L	C
Khan 2022	C	M	L	L	L	L	L	C
Lapointe 2022	S	L	L	L	L	L	L	S
Lombardi 2022	C	M	L	L	L	L	L	C
Loubet 2022	C	L	L	L	L	L	L	C
Lv 2022	C	L	L	L	L	L	L	C
Nault 2022	C	M	L	L	L	L	L	C
Netto 2022	C	L	L	L	L	L	L	C
Oyaert 2022	C	M	L	L	L	L	L	C
Polvere 2022	C	L	L	L	L	L	L	C
Portillo 2022	C	M	L	L	L	L	L	C
Schmidt 2022	C	L	L	L	L	L	L	C
Spinelli 2022	M	L	L	L	L	L	L	M
Tan 2022	C	L	L	L	L	L	L	C
Vergori 2022	C	L	L	L	L	L	L	C
Wong 2022	S	L	L	L	L	L	L	S
Xu 2022	C	L	L	L	L	L	L	C
Zeng 2022	C	L	L	L	L	L	L	C
Zou 2022	C	L	L	L	L	L	L	C

Supplementary Table S7. Meta-regression for seroconversion rate in patients living with HIV after complete vaccination.

**Variable**	**β**	**95% LCI**	**95% UCI**	**P**
**Year of publication**	-0.1634	-0.3719	0.0452	0.1247
**Study location**				
Europe	0.2736	0.1559	0.3912	< .0001
North America	0.0838	-0.1263	0.2939	0.4345
South Africa	-0.2066	-0.4831	0.0699	0.1431
Asia	-0.2653	-0.4240	-0.1065	0.0011
South America	-0.3695	-0.6846	-0.0544	0.0215
**Study design**	0.0680	-0.0832	0.2191	0.3779
**Source of data**	0.1186	-0.0248	0.2620	0.1050
**Sample Size**	-0.0392	-0.1805	0.1021	0.5869
**Follow-up duration**	0.0785	-0.0713	0.2282	0.3043
**Adjust**	-0.0010	-0.1726	0.1705	0.9907
**Antiretroviral therapy**	-0.0590	-0.2638	0.1458	0.5724
**COVID-19 history**	0.1261	-0.0593	0.3114	0.1824
**Vaccine type**				
mRNA vaccine	0.2845	0.1477	0.4213	< .0001
Adenovirus vaccine	0.0533	-0.2341	0.3408	0.7160
Inactivated Vaccine	-0.4460	-0.5808	-0.3113	< .0001
Others	-0.0093	-0.2080	0.1895	0.9270

β: regression coefficient; LCI: lower confidence interval; UCI: upper confidence interval.

Supplementary Table S8. Meta-regression for risk ratio of seroconversion compared with controls after complete vaccination.

Variable	β	95% LCI	95% UCI	P
**Year of publication**	-0.0384	-0.0860	0.0092	0.1136
**Study location**				
Europe	0.0518	0.0090	0.0947	0.0178
North America	-0.0212	-0.0840	0.0416	0.5073
South Africa	-0.0279	-0.1392	0.0834	0.6231
Asia	-0.0205	-0.0760	0.0350	0.4693
South America	-0.1998	-0.3164	-0.0833	0.0008
**Study design**	0.0487	-0.0144	0.1117	0.1302
**Source of data**	0.0355	-0.0085	0.0796	0.1139
**Sample Size**	0.0028	-0.0390	0.0446	0.8954
**Follow-up duration**	0.0037	-0.0409	0.0483	0.8710
**Adjust**	0.0021	-0.0436	0.0477	0.9291
**Antiretroviral therapy**	-0.0211	-0.0769	0.0346	0.4574
**COVID-19 history**	0.0287	-0.0215	0.0790	0.2628
**Vaccine type**				
mRNA vaccine	0.0420	-0.0029	0.0870	0.1383
Adenovirus vaccine	0.0633	-0.0118	0.1383	0.0987
Inactivated Vaccine	-0.2117	-0.2938	-0.1295	< .0001
Others	-0.0108	-0.0658	0.0442	0.7003

β: regression coefficient; LCI: lower confidence interval; UCI: upper confidence interval.

Supplementary Table S9. Certainty of evidence and summary effect estimates assessed by GRADE (grading of recommendations, assessment, development, and evaluation) of the study outcomes.

(a) Meta-analysis of observational studies assessing serological response after vaccine.

Outcomes	Starting level of evidence	Quality assessment$					Reasons to increase level of evidence*	Overall quality of evidence
		Risk of bias	Inconsistency	Indirectness	Impression	Publication bias		
Overall serological response after incomplete vaccination	Low	Serious	Serious	Not serious	Serious	Not serious	Dose-response gradient	Very low
Overall serological response after full vaccination	Low	Serious	Serious	Not serious	Not serious	Serious	Dose-response gradient	Very low
Overall serological response after booster vaccination	Low	Serious	Not serious	Not serious	Not serious	Not serious	Dose-response gradient	Low

$ Quality assessment to decrease level of evidence include risk of bias (≥25% of contributing studies were assessed as serious risk of bias), inconsistency (substantial between study heterogeneity, $I^2 \geq 50\%$), indirectness (presence of factors limiting generalizability of results), impression (95% confidence intervals for risk estimates are wide above 10% for outcomes), and publication bias (evidence of small study effects).

*Reasons to increase level of evidence include large magnitude of effect (risk estimates less than 0.5), dose-response gradient or attenuation of the pooled risk estimates by plausible confounders.

(b) Meta-analysis of observational studies comparing serological response after vaccine to control.

Outcomes	Starting level of evidence	Quality assessment$					Reasons to increase level of evidence*	Overall quality of evidence
		Risk of bias	Inconsistency	Indirectness	Impression	Publication bias		
Overall serological response after incomplete vaccination	Low	Serious	Serious	Not serious	Serious	Serious	Dose-response gradient	Very low
Overall serological response after full vaccination	Low	Serious	Serious	Not serious	Not serious	Serious	Dose-response gradient	Very low
Overall serological response after booster vaccination	Low	Not serious	Not serious	Not serious	Not serious	Not serious	Dose-response gradient	Moderate

$ Quality assessment to decrease level of evidence include risk of bias (≥25% of contributing studies were assessed as serious risk of bias), inconsistency (substantial between study heterogeneity, $I^2 \geq 50\%$), indirectness (presence of factors limiting generalizability of results), impression (95% confidence intervals for risk estimates are wide than 10% for outcomes), and publication bias (evidence of small study effects).

*Reasons to increase level of evidence include large magnitude of effect (risk estimates above 2 or less than 0.5), dose-response gradient or attenuation of the pooled risk estimates by plausible confounders.

Supplementary file

Supplementary File S1. List of excluded studies (n = 13).

Failing to report HIV seropositivity for COVID-19 vaccine (n = 6)

1. Chammartin F, Kusejko K, Pasin C, et al. Determinants of antibody response to severe acute respiratory syndrome coronavirus 2 mRNA vaccines in people with HIV. AIDS. 2022;36(10):1465-1468. doi:10.1097/QAD.0000000000003246
2. Healy K, Pin E, Chen P, et al. Salivary IgG to SARS-CoV-2 indicates seroconversion and correlates to serum neutralization in mRNA-vaccinated immunocompromised individuals. Med (N Y). 2022;3(2):137-153.e3. doi:10.1016/j.medj.2022.01.001
3. Woldemeskel BA, Karaba AH, Garliss CC, et al. Decay of coronavirus disease 2019 mRNA vaccine-induced immunity in people with HIV. AIDS. 2022;36(9):1315-1317. doi:10.1097/QAD.0000000000003263
4. Ogbe A, Pace M, Bittaye M, et al. Durability of ChAdOx1 nCoV-19 vaccination in people living with HIV. JCI Insight. 2022;7(7):e157031. Published 2022 Apr 8. doi:10.1172/jci.insight.157031
5. Chantasrisawad N, Puthanakit T, Tangsathapornpong A, et al. Immunogenicity and Reactogenicity of mRNA BNT162b2 COVID-19 Vaccine among Thai Adolescents with Chronic Diseases. Vaccines (Basel). 2022;10(6):871. Published 2022 May 29. doi:10.3390/vaccines10060871
6. Shinde V, Bhikha S, Hoosain Z, et al. Efficacy of NVX-CoV2373 Covid-19 Vaccine against the B.1.351 Variant. N Engl J Med. 2021;384(20):1899-1909. doi:10.1056/NEJMoa2103055

Cross-sectional study (n = 5)

1. Cai S, Liao G, Yu T, et al. Immunogenicity and safety of an inactivated SARS-CoV-2 vaccine in people living with HIV: A cross-sectional study. J Med Virol. 2022;94(9):4224-4233. doi:10.1002/jmv.27872
2. Wu S, Zou S, Ming F, et al. Humoral Immune Response to Inactivated COVID-19 Vaccination at the 3rd Month among People Living with HIV. Preprint. Res Sq. 2022;rs.3.rs-1750225. Published 2022 Jun 27. doi:10.21203/rs.3.rs-1750225/v1
3. Huang X, Yan Y, Su B, et al. Comparing Immune Responses to Inactivated Vaccines against SARS-CoV-2 between People Living with HIV and HIV-Negative Individuals: A Cross-Sectional Study in China. Viruses. 2022;14(2):277. Published 2022 Jan 28. doi:10.3390/v14020277
4. Yan Y, Davgadorj C, Lyu C, Zhang S, Qiu Y. Immunogenicity of a third dose of inactivated COVID-19 vaccine in people living with HIV-1, HBV, and tuberculosis during the Omicron variant epidemic: A cross-sectional study. J Infect. 2022;85(4):e109-e111. doi:10.1016/j.jinf.2022.06.032
5. Liu Y, Han J, Li X, et al. COVID-19 Vaccination in People Living with HIV (PLWH) in China: A Cross Sectional Study of Vaccine Hesitancy, Safety, and Immunogenicity. Vaccines (Basel). 2021;9(12):1458. Published 2021 Dec 9. doi:10.3390/vaccines9121458

Review (n = 2)

1. Gianserra L, Donà MG, Giuliani E, et al. High seroconversion rate after vaccination with mRNA BNT162b2 vaccine against SARS-CoV-2 among people with HIV - but HIV viremia matters?. AIDS. 2022;36(9):1319-1320. doi:10.1097/QAD.0000000000003239
2. Wolday D, de Wit TFR. Inactivated SARS-CoV-2 vaccine for people with HIV. EClinicalMedicine.2022;45:101327. Published 2022 Mar 5.doi:10.1016/j.eclinm.2022.101327
